# The role of salicylic acid in modulating phenotyping in spring wheat varieties for mitigating drought stress

**DOI:** 10.1186/s12870-024-05620-5

**Published:** 2024-10-11

**Authors:** Rawan A. Awadalla, Ahmed Sallam, Andreas Börner, Maha M. Elshamy, Yasmin M. Heikal

**Affiliations:** 1 Botany Department, Faculty of Science, Mansoura University, Mansoura, 35516 Egypt; 2 Department Genebank, Resources Genetics and Reproduction, Leibniz Institute of Plant Genetics and Crop Plant Research (IPK), Corrensstr. 3, OT Gatersleben D, Stadt Seeland, 06466 Germany; 3 Department of Genetics, Faculty of Agriculture, Assiut University, Assiut, 71526 Egypt

**Keywords:** *Triticum aestivum*, Heritability, Phenotyping, Water- deficit tolerance, Salicylic acid

## Abstract

Climate change-related droughts that recur frequently are one of the biggest obstacles to wheat (*Triticum aestivum* L.) productivity. Worldwide, attempts are being done to establish drought-resistant cultivars. However, progress is slow since drought tolerance is a complex trait controlled by numerous genes, and its expression is influenced by the environment. Phenotypic, biochemical physiological, and genotyping approaches are highlighted as critical research components for leveraging genetic variation in eight wheat genotypes. Treatments included eight spring wheat genotypes (IPK_040, IPK_046, IPK_050, IPK_071, IPK_105, WAS_007, WAS_024 and WAS_031), normal irrigation (NI), drought stress (D) (30% field capacity (FC)), normal irrigation with 0.5 mM SA (NSA), and drought treated with SA (DSA). The results revealed that there was a reduction in relative water content, an increase membrane leakage, and leaf chlorophyll content under drought stress. SA induced the defense responses against drought by increasing the osmolytes and the antioxidative enzymes activities. Compared to the NI group, the DSA treatment improved the water regulation, antioxidant capacity, and drought stress resistance. SA significantly reduced the deleterious effects of water stress on phenotyping more in WAS_ 024 and IPK_ 105 genotypes. The most responsive genotypes to salicylic acid were IPK_ 046 among the IPK genotypes, whereas WAS_031 genotype was amongst WAS genotypes based on the morpho-physiological traits. The findings of this study give a solid foundation for assessing drought resistance in *T. aestivum* and developing cultivation-specific water management methods.

**Graphical Abstract:**

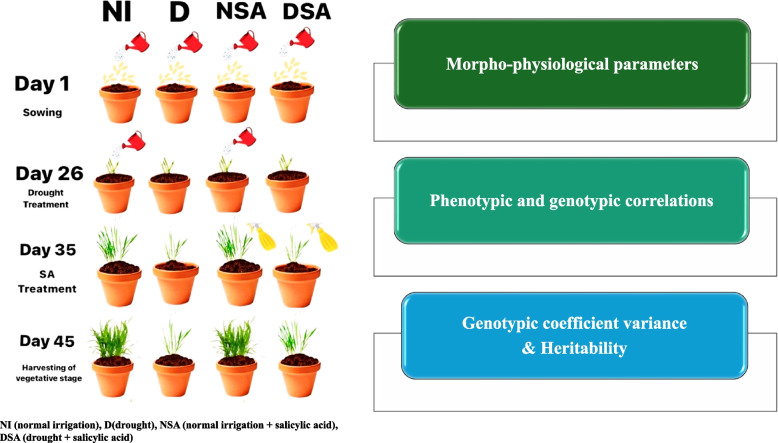

**Supplementary Information:**

The online version contains supplementary material available at 10.1186/s12870-024-05620-5.

## Introduction

Wheat (*Triticum aestivum* L.) is a vital crop in the globe because it includes starch, protein, and sugar and supplies nourishment for the human population [1]. Wheat is one of the most widely cultivated cereal grains, accounting for 17% of the world’s cultivated area and providing a main diet for 35% of people worldwide. More than any other crop, it provides more calories and protein to the world’s diet [2]. Furthermore, grains include trace amounts of lipids, fiber, minerals, and B-group vitamins [3]. Wheat demand will reach 40% in 2030, according to Dixon [4]. Drought is the most critical abiotic stress that impacts practically every element of plant growth through variations in metabolism and gene expression [5]. One of the most harmful environmental pressures is drought, which lowers crop plant output globally. In the world’s major wheat belts, recurrent drought conditions might reduce yield by 23 to 27% by 2050, according to the International Maize and Wheat Improvement Center https://www.cimmyt.org/work/wheat-research/(CIMMYT) [6]. Reactive oxygen species (ROS) build up because of the many physiological changes and metabolic process deficits brought on by drought stress [7–9]. We choose wheat for the experiments because it is one of the most important crops in terms of human consumption and is under threat from climate change and drought. With the aim that our research may illuminate the dark road of climate change.

Plants utilize their drought response mechanisms, which include morphological and structural alterations, the activation of genes resistant to drought, hormone synthesis, and substances that regulate osmotic flow, to lessen the effects of drought stress [10]. Drought stress has a significant impact on wheat plants, including changes in protein, chlorophyll content, growth inhibition, and water content [8, 11]. Furthermore, it influences plant height as well as yield parameters as grain weight, grain yield, and spike length [12, 13]. Plants that are stressed by drought initially show changes in their internal structure and external appearance. The plant grows slowly and eventually dies as the main result of water loss. Research indicates that morphological adaptation is one way in which plants under abiotic stress can adjust to changing environmental conditions. Plant leaves frequently adopt decreased leaf areas, increased leaf thickness, and increased leaf tissue density in response to dryness [14]. One of the most visible characteristics of drought-stressed plant leaves is a decrease in leaf area, which has a direct impact on photosynthesis and output. Previous studies have revealed that leaf turgor pressure, canopy temperature, and photo assimilate availability are the main factors influencing changes in plant leaf area [15]. Wheat cultivars have adapted a variety of drought tolerance mechanisms, including deeper roots, increased biomass, improved stomatal control over transpiration [16], enhanced osmoprotective and antioxidant responses [17], and most importantly, improved coordination of positive and negative gene expression regulation. As a result, wheat’s drought resistance must be improved.

A variety of agronomic and physiological strategies have recently been implemented as complementary approaches to decrease crop losses caused by water scarcity. Plants, fortunately, have multiple complex and well-organized mechanisms in place to buffer the negative impacts of various abiotic stresses. One of the most prevalent of these processes is the ability of plants to biosynthesize and accumulate diverse suitable osmolytes. Plants typically accumulate various compatible osmolytes under stressful conditions cell turgor, protect cellular machinery by allowing continuous water uptake at low soil water potential from stressors, eliminate excess ROS, boost antioxidant enzyme activity, and protect proteins and biological membranes [18, 19]. Salicylic acid (SA), for example, is found in most plants; nevertheless, quantities vary greatly between species [20].

Salicylic acid (SA), a phytohormone, is a potential molecule that can lower plant sensitivity to environmental challenges by controlling the antioxidant defense system, transpiration rates, stomatal movement, and photosynthetic rate [21]. Following this initial characterization, other papers have highlighted the significance of SA as a phytohormone, as well as its critical contribution and diverse role in plants in improving their performance under abiotic stress conditions. SA foliar spray enhances leaf area and light interception, boosting net photosynthesis and grain yield [22]. SA-treated water-stressed wheat plants included more carbohydrates, protein, and minerals [23], as well as solutes (organic and inorganic) [24]. SA successfully enhanced shoot dry matter, hence increasing net CO_2_ assimilation rate. SA improved carbon metabolism, antioxidant system, membrane stability, osmoprotection, and photosynthetic pigments in wheat [25], barley [26, 27], rice [28], maize [29], and tomato [30].

Previous studies demonstrated that low SA concentrations boost plant antioxidant capacity, whereas high SA concentrations cause cell death or sensitivity to abiotic stressors [31]. SA stimulates several genes that encode antioxidants, chaperones, and heat shock proteins. These genes also contribute to the formation of secondary metabolites such as cinnamyl alcohol dehydrogenase, cytochrome P450, and sinapyl alcohol dehydrogenase [32]. Endogenous SA levels in plants rise in response to a water deficit, according to studies [26, 33]. Numerous investigations have demonstrated the positive effects of SA on plants’ responses to abiotic stressors like ozone and ultraviolet (UV) radiation [34, 35], heat stress [36, 37], chilling and drought [36]**,** and salt and osmotic stresses [38].

The current study attempted to (1) gain a better knowledge of the influence of SA on drought stress by evaluating the biochemical and physiological reactions of wheat ecotypes under drought stress and normal conditions, and (2) genetically select the most promising drought-tolerant genotypes associated with morpho-physiological traits under drought stress to be integrated into future breeding programs. With the foregoing context, the objective of the research described here was to gain a better understanding of how exogenous phytohormone (SA) treatment affects wheat cultivars that are drought susceptible or tolerant in both normal and stressed drought scenarios.

## Materials and methods

### Plant materials

Table 1 displayed the eight genotypes of wheat from six different countries, including Egypt, which were chosen based on the selection of Sallam et al., [39] of five susceptible genotypes in addition to three Egyptian genotypes to cover varieties of different genetic origins. The genotypes were obtained from IPK Gatersleben Genebank (http://www.ipk-gatersleben.de/en/genebank/), Germany.

**Table 1:** Genotype name, botanical name and origin of the studied eight spring wheat genotypes

Code	Genotype name	Botanical name	Origin
IPK_040	TRI 4113	*T. aestivum* L. var. *ferrugineum*	Afghanistan
IPK_046	TRI 3633	*T. aestivum* L. var. *lutescens*	Canada
IPK_050	TRI 3564	*T. aestivum* L. var. *ferrugineum*	Portugal
IPK_071	TRI 4940	*T. aestivum* L. var. *aestivum*	USA
IPK_105	TRI 4126	*T. aestivum* L. var. *milturum*	Italy
WAS_007	MISRI1	*T. aestivum* L. var. *aestivum*	Egypt
WAS_024	QDRIY 005	*T. aestivum* L. var. *aestivum*	Egypt
WAS_031	Sohg-5	*T. aestivum* L. var. *aestivum*	Egypt

### Experimental design

This investigation followed a completely randomized design (CRD). Field experiments were conducted at the experimental greenhouse, Faculty of Science, Mansoura University, Mansoura, Egypt. Three replications of each treatment will be used in the experiment, with twelve seeds from each *T. aestivum* genotype being put in pots. The soil in pots was filled with 9 kg of clay and sand (1:1, respectively). The genotypes will be firstly irrigated after sowing with 1.5 L (100%) soil water capacity. This experiment was conducted under four treatment classifications: normal irrigation (NI), drought (D), normal irrigation with salicylic acid (NSA), and drought treated with salicylic acid (DSA). At seven leaf stage of 45 days after sowing (DAS), plants were treated with salicylic acid at 0.5 mM (controls were untreated) under drought stress conditions (30% of soil water capacity). Field capacity (FC) is the amount of water a soil can hold after it has been fully saturated and allowed to drain for a specified period (usually 1–2 days) and it equals 1.5 L (100%); so, 30% of 1.5 L is 0.45 L which used for irrigation under drought stress. The experiment was replicated three times to make a total of 96 pots. Temperature and humidity data were recorded during the experiment in which the temperature ranged from (20—25 °C) and air humidity ranged from 50 to 59%. On the greenhouse, fertilizers in the form of 5% Calcium and Phosphorus, 0.57% Magnesium and 1% Sulphur, the full dose was applied at sowing. Soil was sterilized for controlling fungal and bacterial contamination to avoid diseases such as rust.

### Phenotyping-based genetic variation evaluation

#### Morphological traits of vegetative stage

After 45 days after sowing, all plants of each *T. aestivum* genotype were carefully cleaned from the soil. Fresh weight was measured then dried in oven at 60 ºC for 48 hours then dry weight. For shoot and leaf phenotypic traits: shoot length (ShL), leaf area (LA) and no. of leaves (No. L) were measured and root length (RL) and no. of root (No. R) for root traits. Different leaf and root traits were scanned and measured using image J (version1.50i) software.

### Physiological studies

#### Determination of relative water content (RWC)

For measuring relative water content, the method of Weatherley [40], following the considerations given by El-Sharkawi and Salama [41]. Leaf discs were punched from the center of the leaf. They were weighed to estimate their fresh mass (FM), floated for 4 h on distilled water at 20 °C and weighed again to estimate their turgid mass (TM). For dry mass (DM) determination, the discs were oven dried at 80 ºC for 24 h. Relative water content was calculated as:

$$\mathrm{WC}\left(\%\right)\;=\left(\mathrm{FM}\;-\;\mathrm{DM}\;/\;\mathrm{TM}\;-\;\mathrm{DM}\right)\;\times\;100$$

#### Membrane electrolyte leakage

Membrane electrolyte leakage (ML) was assessed in 10 fully expanded uppermost leaves of 45 days after sowing. A total of ten 1 cm^2^ discs were gathered and cleaned using distilled water. The obtained samples were placed in test tubes with 10 mL of distilled water [42]. The percentage of ML was measured by the following formula:

$$\mathrm{ML}\;=\;\left(\mathrm C1\right)\;/\;\left(\mathrm C2\right)\;\times\;100$$

where C1: EC (electric conductivity) of samples placed in a water bath for 40 °C for 30 min.; C2: EC of the same samples placed in a water bath for 10 min. at 100 °C.

#### Leaf greenness

Leaf greenness (LG) and Chlorophyll Concentration Index (CCI) for each *T. aestivum* leaf sample were measured one time in the middle of leaf by using the Chlorophyll Content Meter CCM-200 plus (Opti-Sciences, Inc., Hudson, NH, USA/Boston, MA, USA). The CCM-200 uses absorbance to estimate the chlorophyll content from the adaxial surface of the leaf tissue via two wavelengths. CCI is the ratio of leaf light transmittance between the wavelength of 931and 653 nm. While one wavelength compensates for mechanical factors such tissue thickness, the other wavelength lies within the spectrum of chlorophyll absorbance. The meter determines the CCI value, which is correlated with the concentration of chlorophyll in the sample, by measuring the absorbance of both wavelengths.

#### Determination of total carbohydrates

Estimates of carbohydrates were made using the protocol of Hedge et al. [43]. Furfural is created when conc. H_2_SO_4_ dehydrates carbohydrates. The enol tautomer of anthrone, anthranol, is the active form of the reagent. It interacts by condensing with the carbohydrate furfural derivative, giving rise to a color that is measured colorimetrically and appears green in diluted solutions and blue in concentrated ones. The blue—green solution shows absorption maximum at 620 nm.

#### Estimation of protein

Scarponi and Perucci [44] method involves homogenizing fresh *T. aestivum* tissue in acetone, followed by sonication. The resulting acetone-dried powder is dried, stored, and used within 24 h. For protein extraction, the powder is mixed with Tris–HCl buffer (0.05 mM, pH 9.0), allowed to stand10 minutes at 4 °C, and then centrifuged at 40,000 xg for 15 min at 4 °C. Protein content is measured spectrophotometrically using Bradford’s [45] method and compared to a standard curve of bovine albumin. The protein concentration is calculated using the formula:

$$\mathrm{Protein}\;=\;\left[{\mathrm{OD}}_{595}\;/\;\mathrm{SL}\right]\;\times\;\left[\mathrm{TV}\;/\;\mathrm{VU}\right]\;\times\left[1\;/\;\mathrm{FW}\right]\;\times\;\left[1\;/\;1000\right]$$

OD = optical density; SL = slope; TV = total volume of the extract (mL); VU = volume used (mL); FW = fresh weight (g).

#### Estimation of non enzymatic antioxidant

The development of the brick-red praline-ninhydrin complex in an acidic media serves as the basis for proline quantification. This complex is soluble in toluene and thus can be separated from aqueous phase. This guarantees that other amino acids, which also combine with ninhydrin to generate a blue-colored complex, won’t interfere. Proline was estimated using a methodology that was substantially outlined by Sunkara et al. [46]. In addition, Baliyan et al. [47] established a technique for measuring the free radical-scavenging activity 2,2-diphenyl-1-picrylhydrazyl (DPPH) of *T. aestivum* methanolic extracts. Using the following formula, the percentage of DPPH scavenging effect was determined.

$$\mathrm{DPPH}\;\mathrm{scavenging}\;\mathrm{effect}\;\left(\%\right)\;=\mathrm A0\;-\;\mathrm A1\;/\mathrm A0\;\times100$$

Where A0 = The absorbance of control and A1 = The absorbance of sample.

#### Estimation of antioxidant enzymes activities

The assay for measuring catalase (CAT) activity was based on Aebi’s [48] methodology. Monitoring the breakdown of H_2_O_2_ at 240 nm allowed for the determination of activity. On decomposition of H_2_O_2_ by catalase, the optical density decreases with time. Also, peroxidase** (**POX) catalyzes the dehydrogenation of a large number of organic compounds such as phenols and aromatic amines. It was determined following the dehydrogenation of guaiacol as a substrate according to Malik and Singh [49]. In addition, polyphenol oxidase (PPO) activity was determined by measuring the initial rate of quinone formation, as indicated by an increase in the absorbance units (AUs) at 420 nm. An increase in absorbance of 0.001 min^−1^ was taken as one unit of enzyme activity [50].

### Statistical analysis

For phenological, physiological, and biochemical parameters among the *T. aestivum* genotypes under different water regimes and salicylic acid application. Using SPSS 16.0 (IBM, Armonk, NY, USA), all collected data were subjected to a two-factor (treatment × genotypes) analysis of variance (ANOVA) using the general linear model. The mean differences were then evaluated using Tukey’s HSD test at *P* ≤ 0.05. Lowercase Latin characters were used to indicate significant changes between genotypes. Three replications of a randomized complete block design were used to obtain the data.

The phenotypic and physiological data were statistically analyzed to estimate variance and covariance using PLABSTAT software [51] and the following statistical model:

$$\mathrm{Yij}\;=\;\mathrm\mu\;+\;\mathrm{gi}\;+\;\mathrm{rj}\;+\;\mathrm{grij}\;\left(\mathrm{error}\right)$$

Where Yij is the observation of genotype i in replication j, is the general average, gi and rj are the genotype and replication main effects, respectively, and the error is the interaction between genotype i and replication j.

Genotypes were regarded as fixed effects, whereas replications were seen as random effects. The HERTI command in PLABSTAT was used to obtain broad-sense heritability (H^2^) estimates for each variable based on genotypic variance (σ^2^G) / phenotypic variance (σ^2^p) for each trait as follows:

$${H}^{2}=\frac{{\sigma }_{G}^{2}}{{\sigma }_{G}^{2}+{\sigma }_{GR}^{2}}$$

where ${\sigma }_{G}^{2}$ is the genotypic variance and ${\sigma }_{G}^{2}+{\sigma }_{GR}^{2}$ is the phenotypic variance.

The phenotypic correlation between traits was calculated using the Spearman rank correlation coefficient. To develop optimal selection indices, the genetic correlation coefficient was computed for all variables using covariance analysis and GENOT- a command in PLABSTAT program.

Cell plots of both morpho-physiological parameters was visualized by JMP®, Version 17.2.0 (SAS Institute Inc., Cary, NC, USA, 2022–2023). Two-way clustering was performed with hierarchical co-clustering, resulting in dendrograms and heatmaps. Row clusters were organized by genotype, and column clusters by trait. Genotypes were grouped based on feature similarity and trait correlation using a clustergram and the Ward method. Additional multivariate analyses, including scatter matrix plots with heatmap correlations, partial correlation diagrams, principal component analysis (PCA), and biplots, were also conducted with the same software.

## Results

### Response of different spring wheat genotypes to the application of salicylic acid under normal irrigation and drought stress at the vegetative stage: phenological markers

#### Phenological and morphological traits characterization

**Fig. 1:**
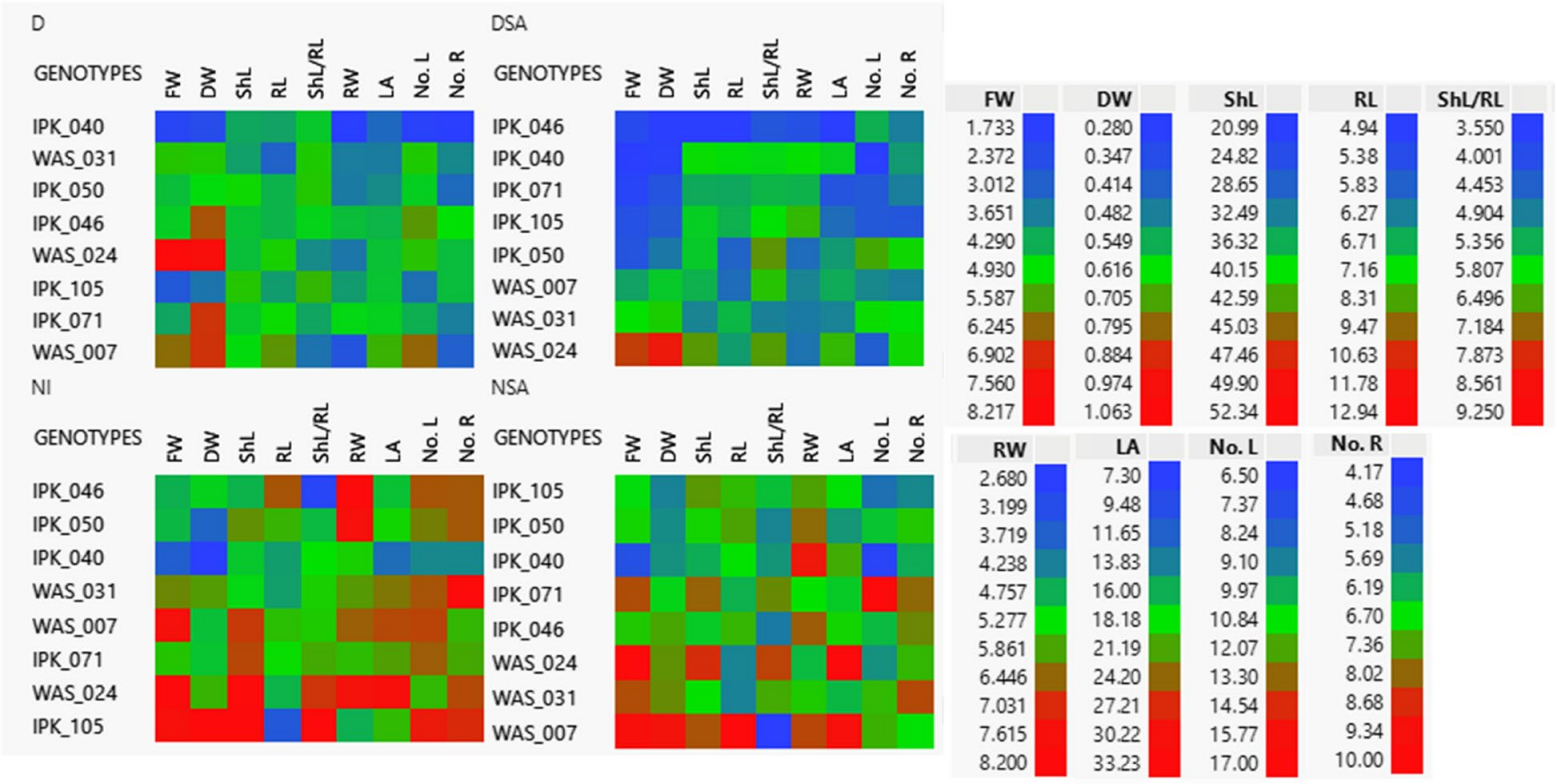
Cell plot of nine quantitative morphological traits of 45 DAS eight spring *Triticum aestivum* under different treatments (D, drought; DSA, drought with salicylic acid; NI, normal irrigation and NSA, normal irrigation with salicylic acid). Abbreviations: FW (fresh weight); DW (dry weight); ShL (shoot length); RW (root width); LA (leaf area); No. L (No. of leaves) and No. R (No. of roots). The red colour indicated the highest values, the green colour indicated moderate values and the blue one indicated the lowest one)

The results of morphological features revealed significant variations between treatments (Fig. 1 and Table 2). These variations are critical for evaluating the effect of salicylic acid on the morphological and phenological characteristics of various genotypes under varied water regimes. Almost all morphological traits showed a highly significant relation between genotyping and treatment interaction (Table 2). Cell plot assessed nine quantitative traits; FW and DW of wheat genotypes in case of NSA was higher than NI. On the other hand, treated plants with SA under drought stress showed lower fresh and dry weight than drought stress without SA application. For shoot phenotypic parameters, shoot length (ShL) means ranged from 36.17 to 43.89 cm, leaf area (LA) means ranged from 14.46 to 21.76 cm^2^ and number of leaves (No. L) was higher in case of normal irrigation and drought stress than of those treated with salicylic acid in both cases. For root traits, root length (RL) varied from 6.4 to 8.1 cm, the mean of plants root width (RW) under DSA was higher than plants under drought stress only. While no. of roots (No. R) of plants under NI was the highest comparing to the other treatments. WAS_024 genotype had the highest value of FW under the four treatments, WAS_007 genotype had higher values in almost all traits under NI and NSA. IPK_105 genotype had high level of traits under NSA than other treatments.

**Table 2:** Two- way ANOVA for morphological traits of 45 DAS eight *T. aestivum* genotypes under different regimes of irrigation and salicylic acid application

Treatments	FW		DW			ShL	RL	(ShL/RL)	RW	LA	No. L	No. R
**Normal Irrigation (NI)**	5.7663 ± 1.87446^c^		0.6138 ± 0.23907^b^			43.894 ± 5.935^c^	7.542 ± 1.336^b^	6.129 ± 1.452^b^	6.554 ± 1.124^b^	21.334 ± 6.165^c^	13.25 ± 1.648^c^	8.083 ± 1.412^c^
**Drought (D)**	4.7346 ± 1.89423^b^		0.7046 ± 0.22895^c^			37.866 ± 2.529^a^	6.795 ± 1.012^a^	5.51 ± 1.029^a^	4.331 ± 0.739^a^	16.36 ± 2.167^b^	10.25 ± 2.436^ab^	5.708 ± 1.122^a^
**Normal + SA (NSA)**	5.8433 ± 1.89723^c^		0.6508 ± 0.17493^bc^			42.039 ± 4.727^b^	8.103 ± 2.288^b^	5.327 ± 1.252^a^	6.387 ± 0.744^b^	21.761 ± 7.132^c^	11.292 ± 3.495^b^	7.042 ± 1.334^b^
**Drought + SA (DSA)**	3.3750 ± 1.69264^a^		0.4946 ± 0.20858^a^			36.175 ± 6.819^a^	6.409 ± 1.037^a^	5.689 ± 0.961^ab^	4.536 ± 0.722^a^	14.465 ± 4.536^a^	9.625 ± 2.584^a^	5.708 ± 1.160^a^
					**Two-way ANOVA**							
**Genotypes (G)**	***		***			***	***	***	**	***	***	***
**Treatments (T)**	***		***			***	***	***	***	***	***	***
Interaction (G* T)	***		***			***	***	***	***	***	***	**

Superscript letters are significant according to the Tukey’s test (*P* ≤ 0.05). Data are represented as mean ± SD and *n* = 3. ***, **, * denote significance at *P* ≤ 0.001,* P* ≤ 0.01, *P* ≤ 0.05, respectively and ns denotes non-significant difference.

*FW* fresh weight,  (g), *DW *dry weight, (g), *ShL *shoot length (cm), *RW *root width, (cm), *LA *leaf area (cm^2^), *No. L, *No. of leaves, *No. R, *No. of roots

#### Phenotypic correlation coefficients

**Fig. 2:**
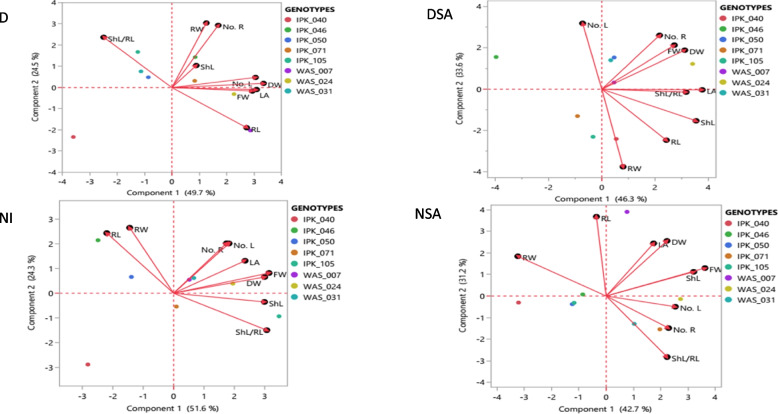
Biplot matrix illustrating the distribution of 45 DAS eight *T. aestivum,* PC1 and PC2 components based on the analysis of morphological traits under different treatments (D, drought; DSA, drought with salicylic acid; NI, normal irrigation and NSA, normal irrigation with salicylic acid). The dots were eight wheat genotypes under different treatments, and the vectors (red arrows) were nine quantitative morphological traits. The abbreviations were previously mentioned in the earlier figures

Figure 2 showed the biplot matrix correlation among nine quantitative morphological traits of 45 DAS eight spring *T. aestivum* genotypes and their distribution under different treatments. Under NI condition, PC1 was 51.6% and the PC2 was 24.3%. IPK_050, IPK_046 and IPK_071 genotypes were closely correlated, and the most frequent vectors differentiated among them were RW and RL. On the other hand, FW, DW, No. R. and No. L vectors differentiated among the rest of genotypes. There was a close strong correlation between FW & DW and No. R. & No. L, while ShL and ShL/RL were negatively correlated to RL and RW.

Under NSA condition, PC1 was 42.7% and the PC2 was 31.2%. RL and RW were the most frequent vectors in differentiating among IPK_050, IPK_046, IPK_105 and IPK_040 genotypes. On the other hand, the other parameters discriminated among WAS_007, WAS_024, and WAS_031 genotypes. RW was negatively correlated to ShL/RL, No. R and No. L. Under D condition, PC1 was 49.7% and the PC2 was 24.5%. FW, LA and DW were the most frequent vectors differentiating among the three WAS genotypes. ShL/RL had a negative correlation with RL which discriminates among IPK_050, IPK_046, IPK_040, WAS_031 and WAS_007 genotypes. Under DSA condition, PC1 was 46.3% and the PC2 was 33.6%. RW was negatively correlated to No. L. which differentiated among IPK_071, IPK_046, IPK_040, and WAS_031 genotypes.

### Physiological and biochemical parameters response of spring wheat varieties under different conditions

#### Water content, leaf greenness, membrane leakage, total carbohydrates, and protein content

**Fig. 3:**
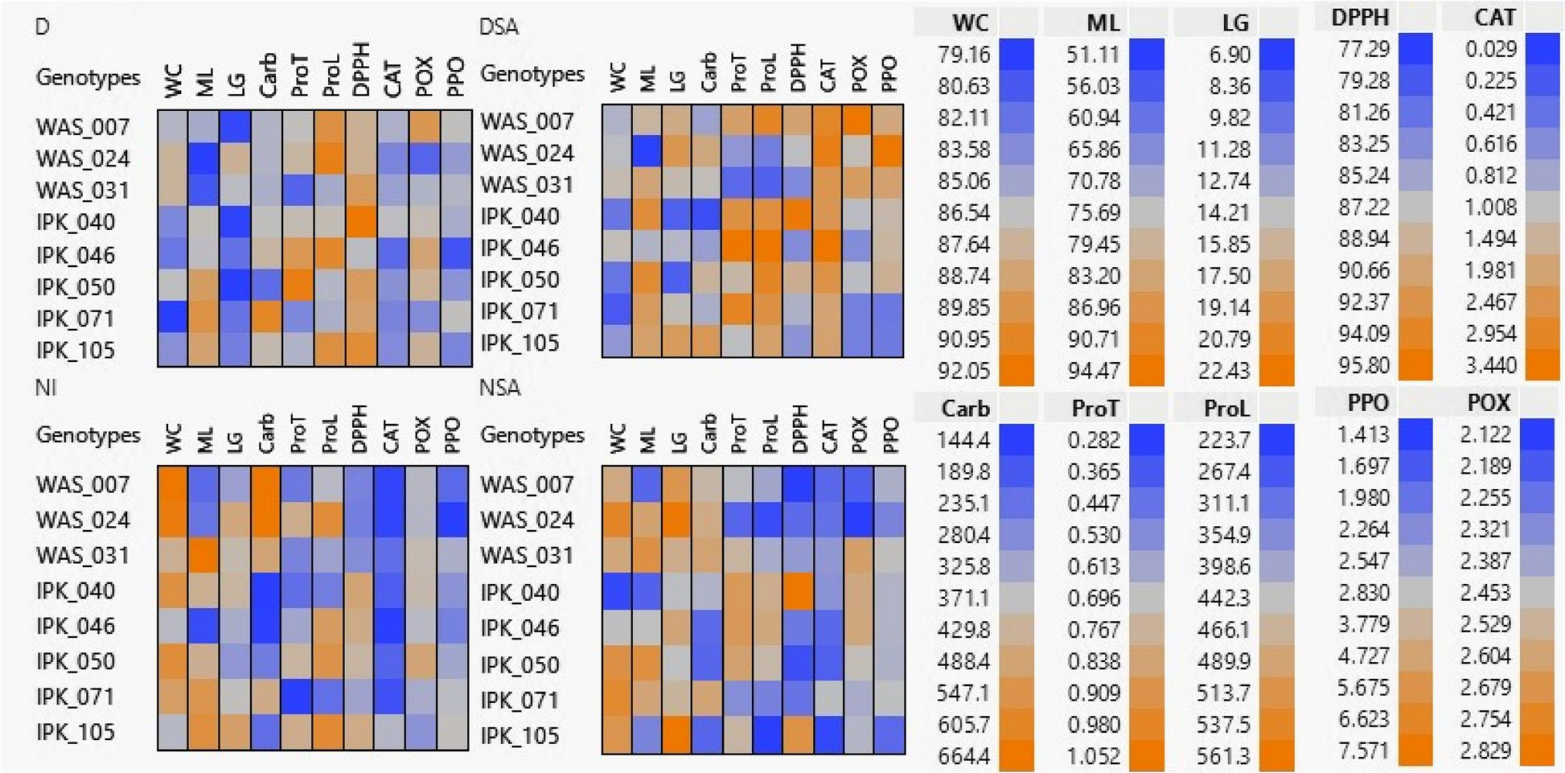
Cell plot of 10 physiological and biochemical traits of 45 DAS eight spring *T. aestivum* under different treatments (D, drought; DSA, drought with salicylic acid; NI, normal irrigation and NSA, normal irrigation with salicylic acid). Abbreviations: WC (water content); ML (membrane leakage); LG (leaf greenness); Carb (carbohydrates); ProT (protein); ProL (proline); CAT (catalase); POX (peroxidase) and PPO (polyphenol oxidase). The orange colour indicated the highest values, while the blue colour showed the lowest ones)

Cell plot of 10 physiological and biochemical traits of 45 DAS eight spring *T. aestivum* under different treatments (Table 3 and Fig. 3). Wheat genotypes showed different performance and physiological response under the influence of the four different conditions in this experiment. The average mean of water content ranged from 84.41 to 89.15 under different circumstances. SA showed a noticeable effect on the IPK_046, IPK_071 and IPK_105 in case of NSA and DSA as they had higher level of water content than their counterparts without SA application. WAS_007, WAS_024 and WAS_031 hardly had significant difference in WC under drought and drought stress treated with SA. Moreover, drought stress reduced chlorophyll and CCI level significantly compared to the control in both untreated *T. aestivum* and SA-treated plants (30.39 and 18.76%), respectively. Foliar application of SA considerably increased chlorophyll content in both well-watered and drought treated plants (17.04 and 28.91%), respectively. There was a substantial difference in chlorophyll content among the eight *T. aestivum* genotypes under both normal and salicylic acid applicated plants. In addition to, drought stress affected membrane electrolyte leakage in all eight genotypes. WAS_031 showed the lowest leakage value under drought conditions, while IPK_050 showed the highest value of ML under salicylic acid treatment in both normal irrigation (NSA) and drought stress conditions (DSA). WAS_024 had the lowest value of leakage under DSA regime. SA showed a remarkable effect on the permeability of WAS_024 and IPK_071; as it reduced the electrolyte leakage by 12.24 and 29.51%, respectively under drought stress conditions (Fig. 3).

**Table 3:** Two- way ANOVA for six physiological and biochemical traits of 45 DAS eight *T. aestivum* genotypes under different regimes of irrigation and salicylic acid application

Treatments	WC	LG	ML	Carb	ProT
**Normal Irrigation (NI)**	89.155 ± 2.164^b^	14.642 ± 2.009^b^	75.525 ± 14.302^ab^	379.616 ± 197.859^a^	0.579 ± 0.176^a^
**Drought (D)**	84.519 ± 2.731^a^	10.192 ± 3.064^a^	73.199 ± 14.58^a^	382.285 ± 105.757^a^	0.706 ± 0.183^b^
**Normal + SA (NSA)**	88.058 ± 3.425^b^	17.65 ± 3.075^c^	74.83 ± 11.978^ab^	353.868 ± 115.088^a^	0.718 ± 0.160^b^
**Drought + SA (DSA)**	84.41 ± 2.275^a^	14.338 ± 3.656^b^	79.216 ± 14.291^b^	368.626 ± 103.14^a^	0.779 ± 0.203^b^
**Two-way ANOVA**					
**Genotypes (G)**	***	***	***	**	***
**Treatments (T)**	***	***	*	**ns**	***
Interaction (G* T)	***	***	***	**ns**	***

Superscript letters are significant according to the Tukey’s test (*P* ≤ 0.05). Data are represented as mean ± SD and *n* = 3. ***, **, * denote significance at *P* ≤ 0.001,* P* ≤ 0.01, *P* ≤ 0.05, respectively and, ns denotes non-significant difference

*WC *water content, (%), *LG *leaf greenness, *ML *Membrane leakage,  (%), *Carb *carbohydrates,  (mg/g), *ProT *protein, (mg/g), *ProL *proline, (mg/g dry wt)

Interestingly, drought stress enhanced the total carbohydrates synthesis of *T. aestivum* under drought stress in both normal drought and drought treated with salicylic acid. Both IPK_046 and IPK_105 showed an increase in total carbohydrates content by 63.94% and 29.43% for IPK_046 and 3.34% and 50.14 for IPK_105, respectively under drought and drought treated with salicylic acid. WAS_031, IPK_040, IPK_046, IPK_071 and IPK_105 genotypes had the highest value of total carbohydrates under normal irrigation treated with SA than in those plants under normal irrigation conditions. As well as protein (ProT) content increased when plants were subjected to drought stress, according to the results in Table 3. Almost all genotypes such as: WAS_007, IPK_040, IPK_046, IPK_050, IPK_050, IPK_071 synthesized high level of protein content under drought stress. IPK_040, IPK_046 and IPK_071 had higher levels of ProT content in both cases of SA application than plants under NI and D conditions. IPK_046 showed the highest protein content under both regulated and stressful circumstances. SA treatment to drought-stressed plants significantly improved soluble protein content compared to plants just treated with drought stress **(**Fig. 3).

### Enzymatic and non -enzymatic antioxidant activity under different water regimes and salicylic acid application

#### Non -enzymatic antioxidant: proline content and DPPH

When plants underwent drought stress, their proline (ProL) content increased under both normal drought and salicylic acid with drought treated *T. aestivum* compared to control in both cases (Table 4 and Fig. 3). ProL in wheat leaves irrigated with salicylic acid under drought was higher than in those watered with fresh water. The majority of genotypes treated with SA showed a higher level of proline content than other circumstances. Likewise, the activity of DPPH ranged from 84.04 to 91.04%. When results are presented graphically as a bar chart, it revealed that DPPH was higher in both cases under D stress conditions than under NI and NSA conditions. WAS_024 had higher levels of DPPH in case of DSA than D treatment. WAS_031, IPK_040 and IPK_105 had high values of DPPH under NSA than under normal irrigation conditions.

**Table 4:** Enzymatic and non -enzymatic antioxidant activity of eight spring wheat genotypes under different water regimes and salicylic acid application

Treatments	Proline	DPPH	CAT	POX	PPO
**Normal Irrigation (NI)**	441.129 ± 83.777^b^	86.446 ± 3.324^ab^	0.28 ± 0.378^a^	2.451 ± 0.071^a^	2.334 ± 0.532^a^
**Drought (D)**	476.882 ± 55.004^c^	91.041 ± 2.515^c^	0.671 ± 0.186^b^	2.471 ± 0.137^a^	2.458 ± 0.470^a^
**Normal + SA (NSA)**	378.226 93.365^a^	84.06 6.250^ab^	0.478 0.316^ab^	2.426 ± 0.169^a^	2.558 ± 0.356^a^
**Drought + SA (DSA)**	473.118 ± 98.632^bc^	87.351 ± 4.071^b^	1.077 ± 0.725^c^	2.466 ± 0.175^a^	3.517 ± 0.943^b^
**Two-way ANOVA**					
**Genotypes (G)**	***	***	**	**	***
**Treatments (T)**	***	***	***	**ns**	***
Interaction (G* T)	***	***	***	*	***

Superscript letters are significant according to the Tukey’s test (*P* ≤ 0.05). Data are represented as mean ± SD and *n* = 3. ***, **, * denote significance at *P* ≤ 0.001,* P* ≤ 0.01, *P* ≤ 0.05, respectively and, ns denotes non-significant difference. DPPH (2,2-diphenylpicrylhydrazyl) (%), CAT (catalase) (mM H_2_O_2_/g. F. wt), POX (peroxidase) (u/g. F. wt/1 min) and PPO (polyphenol oxidase) (u/g. F. wt/5 min)

#### Enzymatic activity

Catalase activity was remarkably increased under DSA in all genotypes than other treatments (Fig. 3) (Table 4). Water-stress induced an increase in CAT activities as it increased under both D and DSA than NI and NSA in all genotypes. Moreover, the mean of peroxidase enzyme activity ranged from 2.426 to 2.471. WAS_007 represented the highest value of POX activity under both D and DSA. IPK_046 represented the highest activity level of POX under NSA, while IPK_050 showed the highest value under NI condition. In addition to, SA showed high PPO activity in almost all genotypes under both condition NSA and DSA. Drought stress increased PPO activity in WAS_031 under both D and DSA conditions. WAS_024 showed the highest value of PPO activity under DSA treatment, while IPK_071 had the highest activity under NSA treatment (Fig. 3).

### Inter- correlation and multivariate analysis based on physiological and biochemical parameters among the studied T. aestivum genotypes

#### Partial correlation

**Fig. 4:**
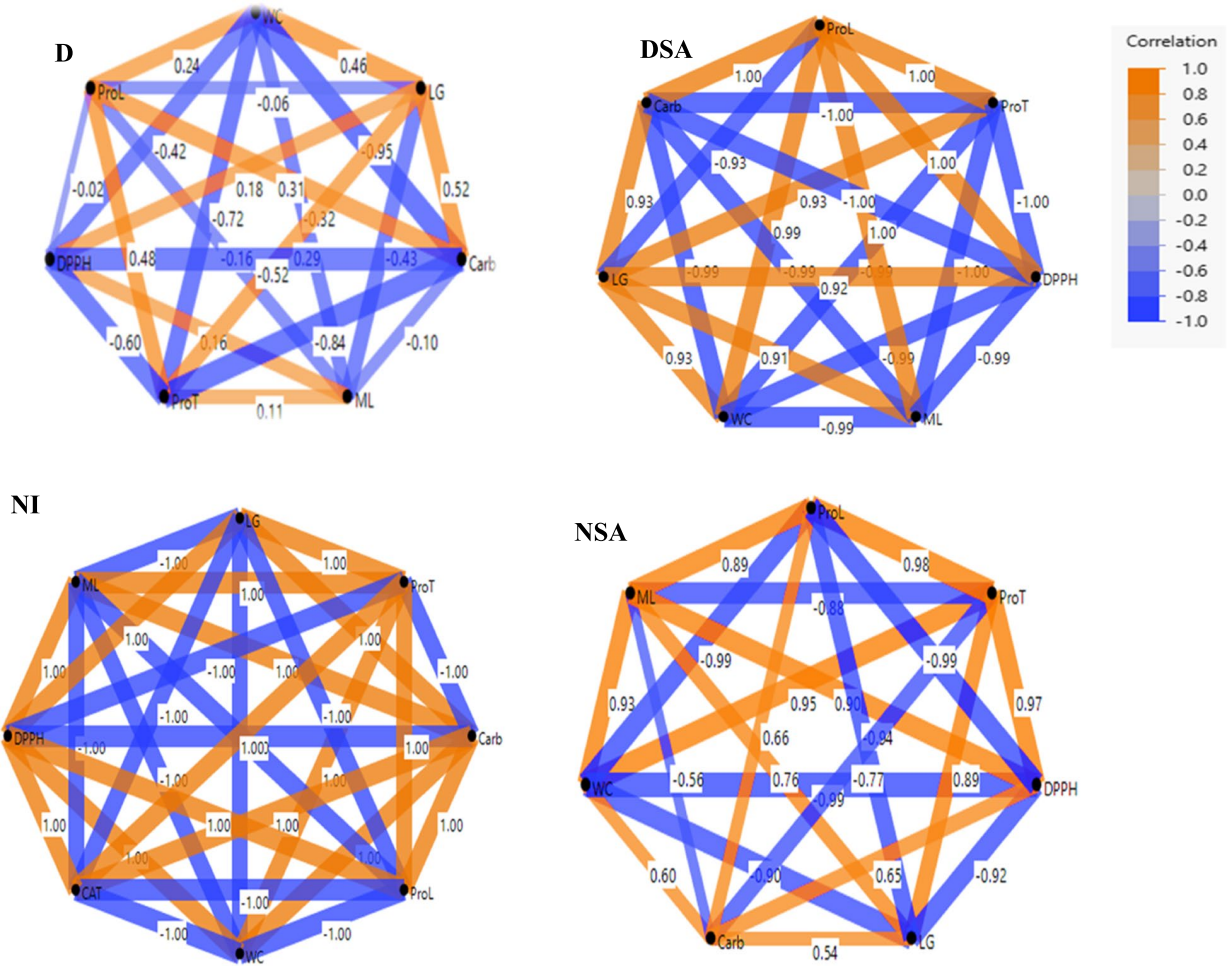
Partial correlation diagram of 10 physiological and biochemical traits of 45 DAS eight spring *T. aestivum* under different treatments (D, drought; DSA, drought with salicylic acid; NI, normal irrigation and NSA, normal irrigation with salicylic acid). Orange colour indicates positive correlation, blue colour indicates the negative correlation, while the thickness of lines indicates the strength of the correlation (see scale at the above right corner)

Figure 4 showed the partial correlation diagram among physiological and biochemical traits of the studied *T. aestivum* genotypes. The orange lines revealed the positive correlation, and the blue one showed the negative ones, while the thickness of lines indicates the strength of the correlation. Under NI condition, WC, LG, ML and ProL contents showed strong positive correlation with ProT, DPPH and Carb contents. After application of SA (NSA), WC had a strong positive correlation with ML, ProT and Carb contents only, while the latter showed a strong positive correlation with WC, LG, DPPH and ProL values. Also, LG, WC, ML, DPPH, ProL and ProT contents revealed high association to each other. Under drought conditions, WC and Carb values had a strong correlation with LG and ProL ones only, while LG and ML contents showed a strong positive correlation with ProT and DPPH contents. Moreover, ProL content had a good association with Carb, WC and ProT contents, while ProT content showed the same correlation to ML and LG contents. After application of SA (DSA), WC, ML, ProT, DPPH and Carb values showed a strong interconnection with LG and ProL contents.

#### Constellation plot

**Fig. 5:**
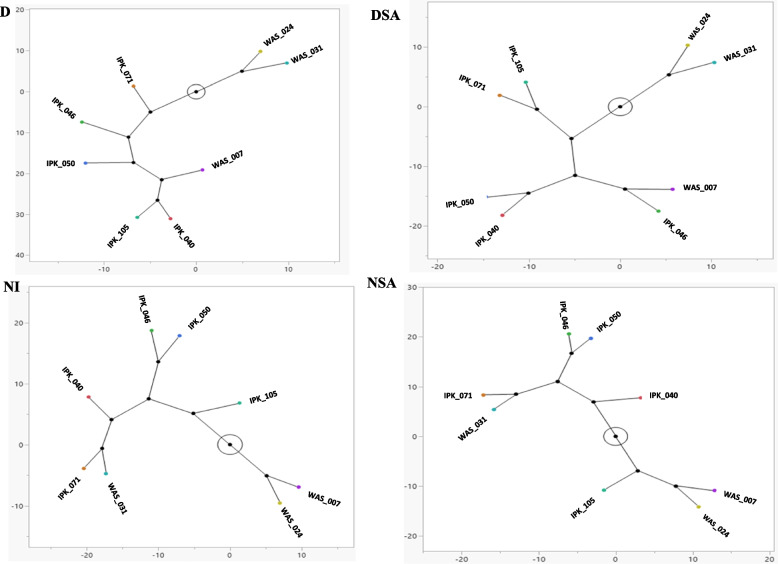
Constellation plot of 10 physiological and biochemical traits of 45 DAS eight spring *T. aestivum* under different treatments (D, drought; DSA, drought with salicylic acid; NI, normal irrigation and NSA, normal irrigation with salicylic acid)

Figure 5 showed the constellation plot and the clustering of 10 physiological and biochemical traits of 45 DAS eight spring *T. aestivum* genotypes under different treatments. Under NI conditions,* T. aestivum* genotypes were divided into two major clusters, the first one had WAS_024 and WAS_007 genotypes. The second cluster divided into 4 sub-clusters, the first subcluster had IPK_105 genotype only, the second sub-cluster had IPK_050 and IPK_046 genotypes, while the third one had IPK_040 genotype only. The fourth sub-cluster had IPK_071 and WAS_031 genotypes. After application of SA (NSA),* T. aestivum* genotypes were divided into two major clusters but IPK_105 genotype moved to the first cluster which had both WAS_024 and WAS_007 genotypes. The rest of *T. aestivum* genotypes persisted as the same clustering under the NI treatment. Under drought conditions,* T. aestivum* genotypes were grouped in two major clusters, WAS_024 and WAS_031 genotypes were found in the first one. Five sub-clusters were formed in the second cluster, each IPK_071, IPK_046, IPK_050 and WAS_007 genotypes had its own sub-cluster. The fifth sub-cluster had both IPK_105 and IPK_040 genotypes. After application of SA (DSA), the two major clusters were divided in a distinct pattern. The first cluster WAS_024 and WAS_031 genotypes, while the second one had only three sub-clusters. IPK_105 and IPK_071 genotypes were detected in the first sub-cluster, while the second one had IPK_040 and IPK_050 genotypes and the third one had WAS_007 and IPK_046 genotypes.

### Inter- correlation and multivariate analysis based on all combined *morpho*-physiological and biochemical traits among the studied T. aestivum genotypes

#### Principal component analysis (PCA) and biplot

**Fig. 6:**
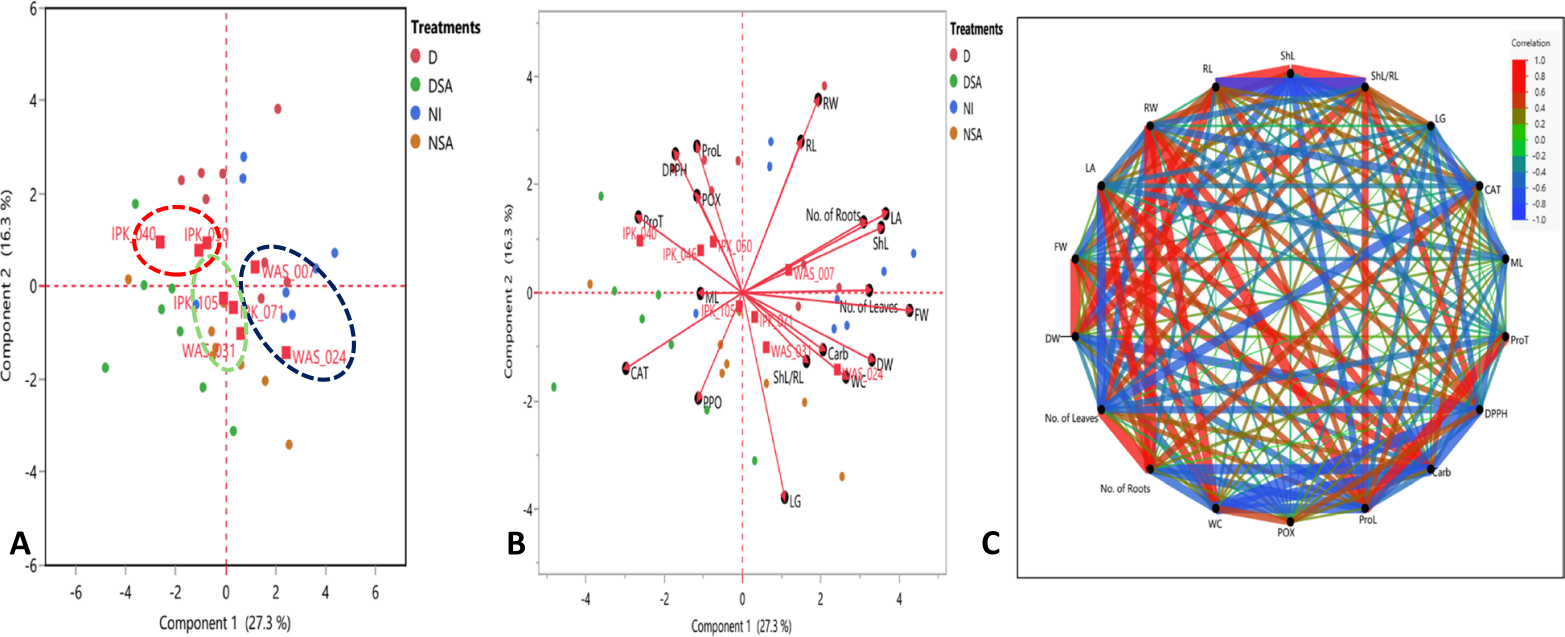
Principal component analysis (PCA), **A** the score plot, **B** biplot and **C** Partial correlation diagram of all 19 combined data (morphological, physiological and biochemical traits) of 45 DAS eight spring *T. aestivum* under different treatments (D, drought; DSA, drought with salicylic acid; NI, normal irrigation and NSA, normal irrigation with salicylic acid). The dashed blue, green and red circles represented the distribution of the genotypes under different conditions. Correlation levels are ranged from blue for negative correlation, green intermediate to red for positive one, while the thickness of lines indicates the strength of the correlation. Abbreviations: FW (fresh weight); DW (dry weight); ShL (shoot length); RW (root width); LA (leaf area); No. L (No. of leaves); No. R (No. of roots); WC (water content); ML (membrane leakage); LG (leaf greenness); Carb (carbohydrates); ProT ( protein); ProL (proline); CAT (catalase); POX ( peroxidase), and PPO (polyphenol oxidase)

Principal component analysis (PCA), the score plot, and biplot of all 19 combined data (morphological, physiological, and biochemical traits) of 45 DAS eight spring *T. aestivum* under different treatments described by the first 2 PCs per cluster as shown in Fig. 6. The first component PC1 was scored as 27.3% and the second component PC2 as 16.3% of the total variation. In a PCA score plot (Fig. 6A), *T. aestivum* genotypes were clustered in three groups. The first blue group had WAS_024 and WAS_007 genotypes under NI condition, while after drought and SA applications the rest of *T. aestivum* genotypes were distributed in the two other groups. The second green group had IPK_105, IPK_071 and WAS_031 genotypes, while the third red group had IPK_040, IPK_046 and IPK_050 genotypes.

Figure 6B showed the biplot in which the system of the two first components, length of vector and cosine of angle were utilized for the discrimination of *T. aestivum* under different treatments. In the first blue group, the most frequent vectors (the longest) were ShL, LA, No. of roots, FW, DW, WC, Carb and ShL/RL. Moreover, LG, PPO and CAT parameters were the most distinctive vectors in discrimination among the *T. aestivum* genotypes in blue and green groups under NSA condition. However, the third red group genotypes were controlled by ProT, POX, ProL, ML and DPPH vectors under drought condition. Also, RL and RW parameters differentiated among the first blue and the third red group genotypes. According to the vector’s correlations, strong positive correlations were found among RW & RL, LA & ShL, Carb &WC & ShL/RL, FW & No. of leaves and POX &ProL & DPPH. On the other hand, negative correlations were detected among RW &RL with PPO, LA & ShL &No. of roots with CAT and DW & Carb & WC & ShL/RL with ProT as shown also in the partial correlation plot (Fig. 6C).

#### Two-way clustering using hierarchical co-clustering dendrogram and heatmap

**Fig. 7:**
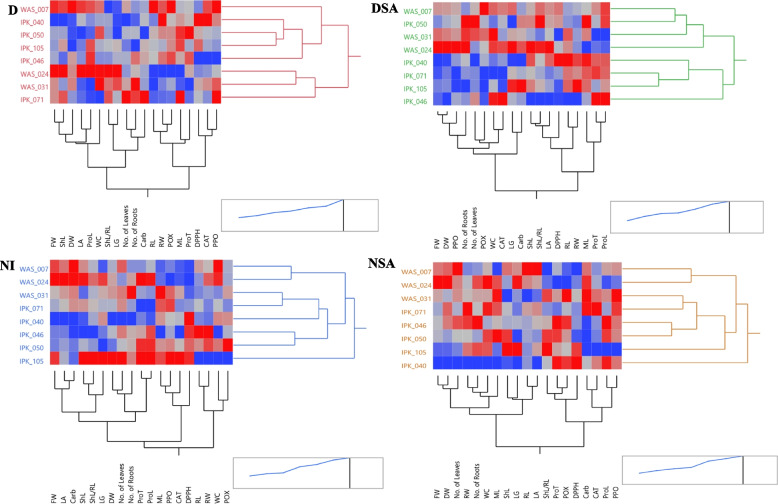
Two-way clustering using hierarchical co-clustering dendrogram and heatmap; row clusters were obtained at genotype level, whereas the column cluster were recorded at trait of all 19 combined data (morphological, physiological and biochemical traits) of 45 DAS eight spring *T. aestivum* under different treatments (D, drought; DSA, drought with salicylic acid; NI, normal irrigation and NSA, normal irrigation with salicylic acid). Correlation levels are colored by red for positive correlation and blue for negative one

Figure 7 showed the two-way co-cluster matrix using hierarchical co-clustering dendrogram using Ward’s method and heatmap correlations; row clusters were obtained at genotype level, whereas the column cluster were recorded at trait or marker of all combined data of submitted to *T. aestivum* genotypes under different treatments. Under NI conditions, *T. aestivum* genotypes were split into two large clusters, the first containing IPK_105 genotype. The second cluster was divided into four subclusters: the first had WAS_024 and WAS_007 genotypes, the second had both the WAS_031 and IPK_071 genotypes, and the third had just the IPK_040 genotype. The fourth sub-cluster contained IPK_050 and IPK_046 genotypes. According to trait or parameter level, the parameters were divided in two main groups. The first one had RW, RL, POX and WC, while the other one had 5 subgroups. The first subgroup had DPPH, CAT, PPO and ML. The second one had ProT and ProL only, while the third one had No. of leaves, No. of roots and DW. Moreover, the fourth one had LG, ShL and ShL/RL, while the last one included FW, LA and Carb.

After application of SA (NSA),* T. aestivum* genotypes were divided into two major clusters but IPK_040 genotype split from other genotypes in a separate cluster. The second cluster was divided into four subclusters: the first had WAS_024 and WAS_007 genotypes, the second had both the WAS_031 and IPK_071 genotypes, and the third contained IPK_050 and IPK_046 genotypes. The fourth sub-cluster had just the IPK_105 genotype. According to trait or parameter level, the parameters were divided in two main groups. The first one had PPO, ProL, CAT, Carb, DPPH, POX, ProT and ShL/RL. While the other one had LA, RL, LG, ShL, ML, WC, No. of roots, RW, No. of leaves, DW and FW.

Under drought conditions, *T. aestivum* genotypes were divided into two large clusters, with WAS_024, WAS_031, and IPK_071 genotypes detected in the first. The second cluster was divided into four sub-clusters, with one for each of the IPK_046, IPK_040, and WAS_007 genotypes. The fourth sub-cluster included both the IPK_105 and IPK_050 genotypes. At parameter level, the parameters were divided in two main groups. The first one had PPO, CAT, DPPH, ProT, ML, POX, RW, RL. While the other one had No. of roots, No. of leaves, Carb, LG, ShL/RL, WC, ProL, LA, DW, ShL and FW.

Following the application of SA (DSA), the two large clusters were separated in a distinct manner. The first cluster had IPK_046 genotype, but the second had five sub-clusters. The first sub-cluster had WAS_007 and IPK_050 genotypes, the second and third had WAS_031 and WAS_024 genotypes, respectively. The fourth one had IPK_040 genotype only, while the last one had IPK_105 and IPK_071 genotypes. At parameter level, the parameters were divided into two main groups. The first one had ProL, ProT, ML, RW, RL, DPPH, LA, ShL, ShL/RL. While the other one had Carb, LG, CAT, WC, POX, No. of leaves, No. of roots, PPO, DW and FW.

### Phenotypic and genotypic correlation coefficients

This study used genotypic (r_g_) and phenotypic (r_p_) correlations to analyze the associations between quantitative characteristics at the phenotypic level (Table 5). The results revealed that the (r_p_) was less than (r_g_) correlation among all phenotypic and physiological traits. FW had the highest correlation in both genotypic and phenotypic traits. FW and DW had the highest correlation ((r_p_) = 0.97 **, *P* < 0.01, (r_g_) = 1.02 + +). Between shoot traits, FW had positive correlation with LA ((r_p_) = 0.86 **, *P* < 0.01, (r_g_) = 0.92 + +). On the other hand, FW had positive correlation with WC ((r_p_) = 0.90 **, *P* < 0.01, (r_g_) = 1.09 + +), CARB ((r_p_) = 0.80*, *P* < 0.01, (r_g_) = 1.04 + +) and PPO ((r_p_) = 0.71*, *P* < 0.01, (r_g_) = 1.07 + +), while had negative correlation with DPPH ((r_p_) = 0.71*, *P* < 0.01, (r_g_) = -0.84 + +).

**Table 5:** Genotypic (rg) (lower diagonal) and phenotypic (rp) (above diagonal) correlation matrix among all morphological, physiological, and biochemical traits of the studied eight T. aestivum genotypes

	**FW**	**DW**	**SHL**	**RL**	**SHL/R**	**RW**	**LA**	**NL**	**NR**	**WC**	**LG**	**ML**	**Carb**	**ProT**	**ProL**	**DPPH**	**CAT**	**POX**	**PPO**
FW	0	0.97**	0.59	0.29	0.45	-0.42	0.86**	0.45	0.37	0.90**	0.7	-0.64	0.80*	-0.49	-0.3	-0.75*	0.58	-0.33	0.71*
DW	1.02 + +	0	0.49	0.3	0.39	-0.32	0.77*	0.54	0.45	0.81*	0.76*	-0.65	0.81*	-0.5	-0.28	-0.81*	0.54	-0.35	0.59
SHL	0.64 + +	0.56 + +	0	0.25	0.68	-0.33	0.78*	0.06	-0.3	0.51	0.51	-0.18	0.59	-0.28	-0.26	-0.19	0.05	-0.57	0.2
RL	0.36	0.37	0.32	0	-0.47	0.35	0.3	0.56	-0.24	0.32	-0.21	-0.17	0.17	0.17	0.46	-0.48	-0.04	0.34	-0.01
SHL/R	0.50 +	0.48 +	0.74 + +	-0.46 +	0	-0.58	0.46	-0.11	0.19	0.39	0.7	0.01	0.51	-0.5	-0.65	-0.03	0.18	-0.68	0.24
RW	-0.80 +	-0.62	-0.67 +	0.42	-1.01 +	0	-0.47	0.19	-0.05	-0.33	-0.41	0.18	-0.63	0.87**	0.89**	-0.05	-0.74*	0.18	-0.7
LA	0.92 + +	0.86 + +	0.80 + +	0.36	0.50 +	-0.95 +	0	0.08	-0.11	0.73*	0.62	-0.71*	0.73*	-0.4	-0.24	-0.41	0.48	-0.45	0.68
NL	0.50 +	0.63 + +	0.06	0.78 + +	-0.17	0.57	0.11	0	0.52	0.43	0.05	0.06	0.45	-0.22	-0.02	-0.88**	-0.01	0.08	0.04
NR	0.41 +	0.53 +	-0.36 +	-0.33	0.2	-0.09	-0.14	0.59 + +	0	0.45	0.32	-0.03	0.17	-0.21	-0.21	-0.6	0.29	0.05	0.26
WC	1.09 + +	1.04 + +	0.62 + +	0.50 +	0.48 +	-0.51	0.92 + +	0.51 +	0.59 +	0	0.48	-0.44	0.55	-0.29	-0.16	-0.68	0.57	-0.05	0.7
LG	0.74 + +	0.85 + +	0.56 + +	-0.3	0.81 + +	-0.80 +	0.67 + +	0.08	0.37 +	0.56 +	0	-0.53	0.62	-0.48	-0.42	-0.31	0.43	-0.62	0.32
ML	-0.76 + +	-0.82 + +	-0.24	-0.15	-0.04	0.7	-0.89 + +	0.07	-0.1	-0.62 +	-0.62 + +	0	-0.41	0.13	-0.07	0.36	-0.5	0.27	-0.62
Carb	1.04 + +	1.13 + +	0.72 + +	0.32	0.65 + +	-1.51 +	0.88 + +	0.59 +	0.22	0.81 + +	0.80 + +	-0.60 +	0	-0.83*	-0.67	-0.61	0.48	-0.49	0.57
ProT	-0.58 + +	-0.61 + +	-0.33	0.29	-0.64 + +	1.84 +	-0.45 +	-0.24	-0.29	-0.35	-0.54 +	0.18	-1.11 + +	0	0.93**	0.26	-0.64	0.25	-0.54
ProL	-0.35	-0.36	-0.31	0.68 +	-0.84 + +	1.85 +	-0.28	-0.06	-0.31	-0.11	-0.46 +	-0.11	-0.98 + +	1.06 + +	0	0.05	-0.5	0.34	-0.43
DPPH	-0.84 + +	-0.95 + +	-0.2	-0.61 +	-0.07	-0.16	-0.46 +	-1.03 + +	-0.72 + +	-0.83 + +	-0.36 +	0.54 +	-0.79 + +	0.3	0.07	0	-0.21	0.12	-0.38
CAT	1.32 +	1.22 +	0.12	0.33	0.25	-1.72 +	1.19 +	-0.19	0.54	1.41 +	1.03	-1.55	1.36 +	-1.60 +	-1.30 +	-0.63	0	0.23	0.83*
POX	-0.44 +	-0.52 +	-0.83 + +	0.65 +	-1.04 + +	0.29	-0.62 +	0.11	0.03	-0.03	-0.87 + +	0.41	-0.90 + +	0.36	0.54 +	0.11	0.34	0	-0.01
PPO	1.07 + +	0.88 + +	0.32	0.02	0.36	-1.53 +	1.05 + +	0.04	0.31	1.20 + +	0.46 +	-1.04 + +	1.11 + +	-0.78 + +	-0.61 +	-0.68 +	1.26 +	-0.19	0

^*^, ** significant at the 0.05 and 0.01 level of the probability, respectively

+ , +  + coefficient of correlation is larger than one and two times the standard error, respectively

DW had positive correlation with LA ((r_p_) = 0.77*, *P* < 0.01, (r_g_) = 0.86 + +), in addition it had positive correlations with WC ((r_p_) = 0.81*, *P* < 0.01, (r_g_) = 1.04 + +), LG ((r_p_) = 0.76*, *P* < 0.01, (r_g_) = 0.85 + +), CARB ((r_p_) = 0.81*, *P* < 0.01, (r_g_) = 1.18 + +) and negative correlation with DPPH((r_p_) = -0.81*, *P* < 0.01, (r_g_) = -0.95 + +). RW had highly positive correlation with ProT ((r_p_) = 0.87**, *P* < 0.01, (r_g_) = 1.84 +), ProL ((r_p_) = 0.89**, *P* < 0.01, (r_g_) = 1.85 +) and negative correlation with CAT ((r_p_) = -0.74*, *P* < 0.01, (r_g_) = -1.72 +). LA had highly positive correlation with WC ((r_p_) = 0.73**, *P* < 0.01, (r_g_) = 0.92 + +) and CARB ((r_p_) = 0.73*, *P* < 0.01, (r_g_) = 0.88 + +) and negative correlation with ML ((r_p_) = -0.71*, p < 0.01, (r_g_) = -0.89 + +). ML had high correlation with DPPH ((r_p_) = -0.88**, *P* < 0.01, (r_g_) = -1.03 + +). CAT had highly positive correlation with PPO ((r_p_) = 0.83*, *P* < 0.01, (r_g_) = 1.26 +).

### Estimation of genotypic coefficient variance, heritability analysis of the studied wheat genotypes

The analysis of mean sum of squares values revealed that genotype variance was significant for all 19 morphological and physiological variables, demonstrating genetic diversity among the eight *T. aestivum* genotypes, as shown in Table 6. Among morphological traits, mean squares of ShL along with other determinants were the highest, while Carb and ProL had the maximum values among different physiological and biochemical traits. The character (LA) had the highest genotypic co-efficient of variation (GCV) (16.963%). Then came the characters ShL, FW, and No. L (13.83, 2.624 and 2.288%), respectively. Amongst the physiological and biochemical parameters, the order of GCV was scored as the following: Carb > ProL > ML > DPPH > LG trait. Heritability (H^2^) ranged from 66.38% to 94.25% for morphological traits. The highest H^2^ values were recorded for FW (94.25%) then came LA (93.87%) and ShL (91.72%), while the lowest was recorded in RL (66.38%). On the other hand, the traits LG, DPPH, ProT showed the highest H^2^ values as (93.89, 85.14 and 78.99%), respectively and the CAT trait had the lowest value (42.81%).

**Table 6:** Estimation of genotypic coefficient variance, heritability analysis of morphological, physiological, and biochemical traits of the studied eight *T. aestivum* genotypes

	**TRAITS**	**R** **MS (2)**	**G** **MS (8)**	**RXG** **MS (16)**	**R** **CV%**	**G** **CV%**	**RXG** **CV %**	**H**^**2**^**%**	**LSD (5%)**	**F value** **of G**
**Morphological traits**	**FW**	0.550	33.407	1.920	-0.0428	2.624	1.920	94.25	1.13	17.40**
	**DW**	0.002	0.254	0.035	-0.0010	0.0183	0.035	86.22	0.15	7.26**
	**SHL**	15.025	180.993	14.983	0.0013	13.834	14.983	91.72	3.14	12.08**
	**RL**	0.766	5.964	2.005	-0.0387	0.33	2.0051	66.38	1.15	2.97**
	**SHL/RL**	0.897	6.098	1.032	-0.0042	0.422	1.0324	83.07	0.83	5.91**
	**RW**	1.149	1.5184	1.101	0.0015	0.0348	1.1012	72.48	0.85	2.83**
	**LA**	8.762	216.841	13.284	-0.1413	16.963	13.284	93.87	2.96	16.32**
	**No. L**	4.041	32.278	4.813	-0.0241	2.2888	4.8138	85.09	1.78	6.71**
	**No. R**	3.135	8.165	1.003	0.0666	0.5968	1.0034	87.71	0.81	8.14**
**Physiological and biochemical traits**	**WC**	2.696	37.834	9.807	-0.2222	2.3356	9.8076	74.08	2.54	3.86**
	**ML**	29.559	820.627	233.403	-6.3701	48.9353	233.40	71.56	12.41	3.52**
	**Carb**	86,995.17	104,762.3	34,405.81	1643.41	5863.03	34,405.82	67.16	150.61	3.04**
	**ProT**	0.260	0.172	0.0363	0.007	0.0114	0.0363	78.99	0.15	4.76**
	**ProL**	9335.37	28,515.64	7040.97	71.6997	1789.55	7040.97	75.31	68.13	4.05**
	**LG**	9.580	90.07	5.500	0.1275	7.0475	5.5005	93.89	1.9	16.37**
	**DPPH**	4.492	133.12	19.782	-0.4778	9.4449	19.782	85.14	3.61	6.73**
	**CAT**	0.166	0.653	0.373	-0.0065	0.0233	0.3739	42.81	0.5	2.15*
	**POX**	0.004	0.092	0.042	-0.0012	0.0042	0.0424	54.22	0.17	2.18*
	**PPO**	0.189	1.789	0.942	-0.0235	0.0706	0.9422	47.34	0.79	2.90**

*R* Replications, *G* Genotypes, *RXG* Replication with genotypes, Parenthesis indicate degree of freedom, *MS* Mean square, *RCV%* Replication Coefficient of Variance, *GCV%* Genotypic Coefficient of Variance, H^2^ (%) heritability, *LSD (5%)* Least significant differences, *F value of G* F value of genotypes, *FW* fresh weight, *DW* dry weight, *ShL* shoot length, *RW* root width, *LA* leaf area, *No. L* No. of leaves, *No. R* no. of roots, *WC* water content, *ML* Membrane leakage, *LG* leaf greenness, *Carb* carbohydrates, *ProT* protein, *ProL* proline, *DPPH* 2,2-diphenylpicrylhydrazyl, *CAT* catalase, *POX* peroxidase, *PPO* polyphenol oxidase

*, ** significant at the 0.05 and 0.01 level of the probability, respectively

## Discussion

Climate change will exacerbate the consequences of drought stress in the agricultural sector by restricting the output and productivity of essential agricultural crops (e.g., wheat). Any stage of growth might experience drought stress in the absence of irrigation. The environment in which the drought occurs determines how severe the drought stress is [52]. Considering the scarcity of water and the extensive use of wheat in the food industry and animal feed, the aim of this study was to ascertain how exogenous salicylic acid levels affected some morpho-physiological characteristics associated with wheat drought tolerance in eight distinct wheat genotypes. Moreover, the genome of wheat is vast and complicated. Therefore, it is necessary to integrate different knowledge systems and methodologies from different plant sciences fields in order to breed for drought tolerance [53]. Different spring wheat cultivars were treated with normal irrigation (NI), drought (D), normal irrigation with salicylic acid (NSA) and drought treated with salicylic acid (DSA) conditions in this investigation and their responses to drought and salicylic acid were carefully evaluated from the perspectives of plant phenotypic, physiological, and genotypic changes.

### Phenological and morphological traits characterization

Vascular plants respond to drought stress through complex regulatory processes involving various morphological and physiological changes [54]. Salicylic acid (SA) is a key phytohormone that manages drought stress by controlling stomatal closure and stress-responsive gene expression. Recent research has identified hormone-like peptides as important signaling molecules that facilitate drought responses by enabling communication between different plant organs [55, 56]. These peptides act as mobile signals within the plant’s vascular system. Studies have also explored how phytohormone signaling, gene expression, and metabolite synthesis are regulated during drought stress. A critical aspect of this response involves redox signaling through SA, which helps manage oxidative stress by reducing the production of reactive oxygen species (ROS), a key indicator of plant tolerance to drought.The connection between SA and ROS in plant signal transduction has been the subject of numerous investigations [57]. The second mechanism involves the involvement of various plant hormones in the SA signal transduction system of plants, in addition to ROS [58]. Under stress, ABA and SA levels have been found to be related in the majority of investigations. The third method involved the regulation of SA levels in stressed plants through the action of mitogen-activated protein kinase (MAPK) [59]. Under an avirulent race of pathogen attack, it was revealed that SA administration enhanced the *TaMAPK4* transcripts in wheat, while *TaMAPK4* gene knockdown downregulated the SA accumulation [60].

Both morphological and phenological parameters, including plant height, biomass, root length, leaf area, number of leaves, and number of roots, were significantly impacted by drought stress. To fend against drought, wheat plants have evolved a variety of tolerance strategies, including escape, avoidance, and tolerance [61]. Two significant factors that are adversely affected by drought stress are plant growth and biomass allocation [62]. According to Kang et al. [63], enhanced carbon absorption and increased plant biomass allow SA to mitigate the effects of drought stress.

In this investigation, fresh weight (FW) in both salicylic acid under NI and D stress conditions had high correlation with other parameters than FW of plants under NI conditions. After SA application, WAS_031 and IPK_050 genotypes had the highest FW under NSA treatments. Sharma et al. [64] study examined two varieties of Triticum aestivum L.: drought-tolerant Kundan and drought-sensitive Lok1 under two distinct water deficit regimes and rehydration during the vegetative and flowering stages. They discovered that the biomass allocation was higher in Kundan’s shoots and lower in Lok1’s roots. El Tayeb and Ahmed [23] described a similar pattern of biomass allocation in drought-stressed, tolerant, and drought-stressed wheat plants treated with SA. Furthermore, a plant can improve the shoot system by increasing the amount of water availability through its roots by growing both in length and number [65].

In this investigation, shoot length (ShL) showed stronger correlations with other parameters under drought stress treated with salicylic acid (SA) compared to other conditions. The number of roots (No. R) had a notable effect among genotypes under drought stress with SA (DSA) rather than under other water regimes [66]. Drought avoidance, a mechanism involving a well-developed root system, was evident as root length (RL) and root width (RW) were higher under DSA than under drought stress alone. RL was a key differentiator among the genotypes WAS_031, WAS_024, and WAS_007 under drought conditions. Partial correlation and biplot analyses revealed a strong positive correlation between No. R and number of leaves (No. L), while ShL and ShL/RL were negatively correlated with RL and RW.

Akram et al. [67] emphasized the importance of root number (RN) and RL in assessing wheat’s tolerance to salinity. Deep root systems help plants avoid drought by accessing moisture from deeper soil layers, enhancing photosynthesis, and increasing the root-to-shoot ratio [68, 69]. According to Reddy et al. [70], temperature, leaf turgor pressure, and assimilation rates affect leaf area (LA). Drought stress led to a decrease in LA in some genotypes (WAS_031, IPK_040, IPK_050), while it increased in WAS_031 under DSA treatment. LA was strongly positively correlated with No. L under drought conditions and showed high correlation with ShL/RL and ShL after SA application. Overall, drought stress significantly impacted morphological traits such as leaf area and plant height in wheat [71].

### Physiological and biochemical traits characterization

Traditional phenotyping procedures for trait quantification and data management are slow, costly, and inconsistent, hindering progress in improving drought tolerance. Mwadzingeni et al. [53] suggested that understanding the genetic and physiological basis of drought tolerance is complicated at both the phenotypic and genomic levels. For physiological and biochemical traits, the eight wheat genotypes showed a wide range of significance under different regimes of irrigation and SA application. The most significant indicator of a plant’s ability to withstand dehydration is its relative water content (RWC), which is a measurement of the water status of the plant and reflects the metabolic activity in tissues. It has been observed that many different types of plants exhibit a reduction in RWC in response to drought stress [72].

In comparison to sensitive cultivars, drought-tolerant cultivars have better water use efficiency; this benefit in drought-tolerant plants may be caused by higher biomass accumulation and reduced evapotranspiration as a result of stomatal closure [73]. In the current study, IPK_046, IPK_071 and IPK_105 genotypes had higher level of WC under NSA and DSA treatments. In addition, WC values had a strong correlation with LG ones under drought conditions. Drought-induced drop in leaf growth also has a negative impact on photosynthesis, as declines in water contents are accompanied by a fall in relative water contents [74]. One essential response of plants to drought stress is to reduce stomatal opening to minimize water loss by transpiration; as a result, CO_2_ diffusion into the leaf is hindered, potentially reducing photosynthesis [75].

In this study, according to two-way clustering analysis, LG, ShL/RL, WC and LA was found in the same cluster and had a positive correlation among them. Also, exogenous SA application mitigated the inhibitory effect of drought stress on the leaf greenness (LG) and photosynthetic capability of wheat genotypes. Similar results have been published on improved photosynthetic rate during drought stress [76, 77]. SA can increase stomatal opening, which improves carbon availability for photosynthesis. SA may also affect metabolic processes involved in carbon uptake and/or fixation in the chloroplast. Furthermore, SA contributes to the integrity of the light-harvesting apparatus [21], a process that may be responsible for increased photosynthesis during drought stress.

According to Guo et al. [78], electrolyte leakage is caused by stress on the plasmalemma. The results suggest that drought stress did cause damages to the membrane system in most of wheat genotypes, which increased plasma membrane electrolytic leakage. In agreement with these previous reports, this result indicated that exogenous SA application improved cell membrane stability. Additionally, Khalvandi et al. [77] findings demonstrated that under drought stress circumstances, there was a clear increase in membrane electrolyte leakage and lipid peroxidation of all six Sardari wheat ecotypes (Kalati, Baharband, Gavdareh, Fetrezamin, Tazehabad, and Telvar).

On the other hand, drought stress decreased leaf chlorophyll content, photosynthetic rate, stomatal conductance, carboxylation efficiency, and transpiration rate. In the current study, lower membrane leakage (ML) in SA-treated plants can be attributed to the protection of cell membrane structure from oxidation, which suggests an improved performance of ROS scavenger activity, such as CAT, PPO, and POX activities. According to biplot, the red and green clusters of genotypes were controlled by ProT, POX, ProL, ML and DPPH vectors under drought condition and after SA-application. In drought-tolerant wheat genotypes under drought stress, Dudziak et al. [79] detected a considerable increase in the expression pattern of genes encoding CAT, APX, and GPX enzymes. These genes may be crucial in regulating the wheat genome’s response to drought stress. Antioxidants prevent free radicals from causing cell damage, which makes them a viable tool for enhancing plant defense mechanisms [80]. The free radical scavenging (DPPH) method is the first way for testing the antioxidant capability of a chemical. The current results revealed that DPPH was higher under D stress conditions than under NI and NSA conditions. Anwar et al. [81] use wheat to assess the effectiveness of GA3-enriched biochar (GA3-BC) against cadmium and drought stress. They discovered that under drought stress, wheat treated with GA3-BC1 showed a 25.06% increase in DPPH activity compared to control.

In response to drought stress, plants produce osmoregulatory compounds like sugars, polyols, and amino acids, which help manage water potential and enhance stress tolerance [82]. According to Chen et al. [25], salicylic acid (SA) triggers various defense mechanisms against oxidative stress, including activating an efficient antioxidant system, osmoregulation, and increasing protective proteins. Exogenous SA application significantly influences the synthesis of stress-related proteins and enhances antioxidant enzyme activity, contributing to drought resistance [83].

Principal Component Analysis (PCA) in this study highlighted the importance of antioxidant systems and defensive proteins in SA-induced drought tolerance, showing strong correlations among several key proteins and enzymes, such as ProT, POX, ProL, DPPH, ML, CAT, and PPO. Sharma et al. [64] also observed that SA increased the activities of antioxidant enzymes like SOD, CAT, APX, and GR during the vegetative stage of wheat, providing drought protection.

Carbohydrates play multiple roles, including carbon storage, detoxification of reactive oxygen species, and stabilization of macromolecules. They are more effective than proline in severely dehydrated conditions due to their ability to provide a hydration shell for proteins [84]. According to Sara et al. [85], protein synthesis can be impaired under drought stress, leading to reduced plant growth and crop yield. Due to the decreased number of polysomal complexes in tissues with lower water content, the reported impaired protein synthesis in this case was accompanied by a loss in plant growth and crop output under water stress conditions [86].

This study found a strong correlation between physiological and biochemical traits (e.g., water content, ML, ProT, DPPH, and carbohydrate levels) with leaf growth and proline content under drought stress with SA treatment. PCA and biplot analyses emphasized proline’s role in SA-induced drought tolerance, showing high values in most genotypes except WAS_031 and WAS_024. Additionally, La et al. [87] found that pre-treatment with SA reduce proline concentration and enhance the expression of redox-regulating genes, thereby mitigating drought-induced oxidative damage.

### Phenotypic and genotypic correlation coefficients between morphological and physiological traits.

The way that genotyping and phenotyping interact with one another, and the environment influences the performance of wheat crops. Since correlations must be based on inherited behavior, genotypic correlation values are required for more research. The pleotropic effects of a gene, chromogema, the coupling of two genes and regimental affiliation, or environmental factors can all result in trait genetic connections [88]. A signal in the altered behavior of breeding germplasm under the cover of different moisture states is the significant Genotype × Water treatments interaction [89]. In the current study, LA had highly positive genotypic association with WC (r_g_) = 0.92 + +) and CARB ((r_g_) = 0.88 + +). Also, RW had highly positive correlation with ProT (r_g_) = 1.84 +) and ProL (r_g_) = 1.85 +). In the same trend, Ahmed et al., 2020 discovered that both water treatments produced considerably different effects, with increases in proline and sugar accumulation in wheat genotypes under the two environmental conditions, optimum water condition (E1) and water shortage condition (E2), of 159% and 122%, respectively. Likewise, under drought conditions, they discovered that there was a stronger genotypic paired association between sugar and the leaf membrane stability index, leaf relative water content, and canopy temperature depression; however, in optimal moisture conditions, there was a strong genotypic association with the relative dry weight, chlorophyll, and leaf surface area.

Heritability, a measure of phenotypic diversity caused by genetics, serves as a predictor in crop breeding [90]. Plant breeding success is determined by the phenotypic and genotypic variability (PCV and GCV) of a crop population. They are critical factors for assessing how the environment influences genotype performance [91]. Plant breeding success depends on the phenotypic and genotypic variations (PCV and GCV) of a crop population. If the PCV and GCV readings are virtually similar or have a very tiny difference, it means that the attribute is predominantly determined by the genotype’s genetic makeup. The bigger the disparity between PCV and GCV levels, the more the environment influences the genotype’s performance. It is an important parameter for assessing how the environment affects genotype performance [92]. In this investigation, ShL, LA, FW, DPPH, LG, WC, Carb and ProL traits showed high GCV and heritability. The high heritability indicated that classification for these features would be effective since it would be less influenced by environmental factors [93]. The simpler the selection techniques, the greater the heritability estimates [94]. Furthermore, Sewore et al. [95] discovered that under well-watered conditions (80% of field capacity), the heritability in broad sense (H2BS) values of one hundred and ninety-six bread wheat genotypes ranged from 43.28% for the relative water loss (RWL) to 85.75% for the anthesis (SPADA) trait, while under drought-stressed conditions (35% of field capacity) a range of 43.02% for trait RWL to 87.29% for the leaf membrane stability index (LMSI) trait.

## Conclusions

Our findings support the hypothesis that wheat’s tolerance to drought is governed by a number of genetic and physiological systems. Salicylic acid treatment may have a key role in controlling the physiological processes that eventually protect wheat from drought stress. SA protected plant biomass, water capacity, and cell membrane integrity while simultaneously increasing ROS scavenger activity (CAT, PPO, and POD). Based on morpho-physiological differences between genotypes in response to drought, it is believed that IPK_050 and WAS_024 are the least genotypes responded to SA treatments, whereas the most responsive genotypes to SA treatments were IPK_046, and WAS_031. In summary, SA seems like a potential method that could be used to mitigate the negative effects of drought stress on wheat in areas with significant water scarcity. It is recommended to use the very promising drought-resistant genotypes that were selected for through genetic means as future parents in crossbreeding.

Using salicylic acid (SA) to enhance drought tolerance in plants has several limitations such as, the impact of SA on drought tolerance can vary widely among different plant species and genotypes, making it difficult to generalize its effectiveness, determining the precise concentration of SA needed is challenging, as too little may be ineffective while too much can be harmful or lead to toxicity, and SA might offer only temporary relief from drought stress rather than long-term drought resilience, necessitating additional management strategies.

Based on the study results, the following recommendations are suggested to conduct further research to explore how different wheat genotypes respond to various environmental conditions and salicylic acid (SA) treatments. This will aid in optimizing SA application strategies and determining best practices for specific environments.

## Supplementary Information

Supplementary Material 1.

## Data Availability

Data is provided within the manuscript or supplementary information files.

## Abbreviations

DAS: Days after sowing
SA: Salicylic acid
NI: Normal irrigation
D: Drought
NSA: Normal + SA
DSA: Drought + SA
ShL: Shoot length
RL: Root length
LA: Leaf area
NO. L: No. of leaves
NO. R: No. of roots
WC: Water content
ML: Membrane leakage
LG: Leaf greenness
Carb: Carbohydrates
ProT: Protein
DPPH: 2,2-Diphenyl-1-picrylhydrazyl
ProL: Proline
CAT: Catalase
POX: Peroxidase
PPO: Polyphenol oxidase
ROS: Reactive oxygen species
CRD: Completely randomized design

## References

[1] Liu X, Zhu X, Pan Y, Li S, Liu Y, Ma Y. Agricultural drought monitoring: Progress, challenges, and prospects. J Geog Sci. 2016;26(6):750–67. 10.1007/s11442-016-1297-9.

[2] Rashid K, Arfan M, Majid M, Ali S, Anwar A, Farhan KM. Response of wheat plant to drought stress: a review. Plant Cell Biotechnology and Molecular Biology. 2021;22(72):263–71.

[3] Roohi E, Mohammadi R, Niane AA, Niazian M, Niedbała G. Agronomic Performance of Rainfed Barley Genotypes under Different Tillage Systems in Highland Areas of Dryland Conditions. Agronomy. 2022;12(5):1070. 10.3390/agronomy12051070.

[4] Dixon J, Braun H-J, Kosina P, and Crouch JH, editors. Wheat Facts and Futures 2009. Mexico, D.F.: CIMMYT International Maize and Wheat Improvement Center. https://ideas.repec.org/p/ags/cimmfa/56366.html.

[5] Seleiman MF, Al-Suhaibani N, Ali N, Akmal M, Alotaibi M, Refay Y, Dindaroglu T, Abdul-Wajid HH, Battaglia ML. Drought Stress Impacts on Plants and Different Approaches to Alleviate Its Adverse Effects. Plants (Basel). 2021;10(2):259. 10.3390/plants10020259. 33525688 10.3390/plants10020259 PMC7911879

[6] Kommerell V, Listman GM. Wheat Matters. International Center for Agricultural Research in the Dry Ares (ICARDA), International Maize and Wheat Improvement Center (CIMMYT). Mexico; 2014. https://repository.cimmyt.org/entities/publication/2202b0a8-5ee7-490b-aa74-45e49ea449bd.

[7] Abid M, Ali S, Qi LK, Zahoor R, Tian Z, Jiang D, Snider JL, Dai T. Physiological and biochemical changes during drought and recovery periods at tillering and jointing stages in wheat (*Triticum aestivum* L.). Sci Rep. 2018;8(1):4615. 10.1038/s41598-018-21441-7. 29545536 10.1038/s41598-018-21441-7 PMC5854670

[8] Lawlor DW, Cornic G. Photosynthetic carbon assimilation and associated metabolism in relation to water deficits in higher plants. Plant Cell Environ. 2002;25(2):275–94. 10.1046/j.0016-8025.2001.00814.x. 11841670 10.1046/j.0016-8025.2001.00814.x

[9] Qaseem MF, Qureshi R, Shaheen H. Effects of Pre-Anthesis Drought, Heat and Their Combination on the Growth, Yield and Physiology of diverse Wheat (*Triticum aestivum* L.) Genotypes Varying in Sensitivity to Heat and drought stress. Sci Rep. 2019;9(1):6955. 10.1038/s41598-019-43477-z. 31061444 10.1038/s41598-019-43477-z PMC6502848

[10] Yang X, Lu M, Wang Y, Wang Y, Liu Z, Chen S. Response Mechanism of Plants to Drought Stress. Horticulturae. 2021;7(3):50. 10.3390/horticulturae7030050.

[11] Yordanov I, Velikova V, Tsonev T. Plant Responses to Drought, Acclimation, and Stress Tolerance. Photosynthetica. 2000;38(2):171–86. 10.1023/A:1007201411474.

[12] Rivero RM, Kojima M, Gepstein A, Sakakibara H, Mittler R, Gepstein S, Blumwald E. Delayed leaf senescence induces extreme drought tolerance in a flowering plant. Proc Natl Acad Sci. 2007;104(49):19631–6. 10.1073/pnas.0709453104. 18048328 10.1073/pnas.0709453104 PMC2148340

[13] Sallam A, Hashad M, Hamed E-S, Omara M. Genetic variation of stem characters in wheat and their relation to kernel weight under drought and heat stresses. J Crop Sci Biotechnol. 2015;18(3):137–46. 10.1007/s12892-015-0014-z.

[14] Werner C, Correia O, Beyschlag W. Two different strategies of Mediterranean macchia plants to avoid photo-inhibitory damage by excessive radiation levels during summer drought. Acta Oecologica. 1999;20(1):15–23. 10.1016/S1146-609X(99)80011-3.

[15] Taiz L, Zeiger E. Plant Physiology and Development; Sinauer Associates: Sunderland. USA: MA; 2015.

[16] Chipilski RR, Kochev KV, Nenova VR, Georgiev GI. Physiological Responses of Two Wheat Cultivars to Soil Drought. Zeitschrift für Naturforschung C. 2012;67:0181. 10.5560/ZNC.2012.67c0181. 22624334

[17] Huseynova IM. hotosynthetic characteristics and enzymatic antioxidant capacity of leaves from wheat cultivars exposed to drought. Biochimica et Biophysica Acta (BBA) - Bioener. 2012;1817(8):1516–23. 10.1016/j.bbabio.2012.02.037. 10.1016/j.bbabio.2012.02.037 22417798

[18] Dutta T, Neelapu NRR, Wani SH, and Challa S. Compatible Solute Engineering of Crop Plants for Improved Tolerance Toward Abiotic Stresses. In. Biochemical, Physiological and Molecular Avenues for Combating Abiotic Stress Tolerance in Plants. (Elsevier), 2018: 221–254. 10.1016/B978-0-12-813066-7.00012-7.

[19] Ghosh UK, Islam MdN, Siddiqui MdN, and Khan MdAR. Understanding the roles of osmolytes for acclimatizing plants to changing environment: a review of potential mechanism. Plant Signal Behav. 2021:16(8). 10.1080/15592324.2021.1913306. 10.1080/15592324.2021.1913306 PMC8244753 34134596

[20] Maruri-López I, Aviles-Baltazar NY, Buchala A, Serrano M. Intra and Extracellular Journey of the Phytohormone Salicylic Acid. Front Plant Sci. 2019;10:423. 10.3389/fpls.2019.00423. 31057566 10.3389/fpls.2019.00423 PMC6477076

[21] Nazar R, Umar S, Khan NA, Sareer O. Salicylic acid supplementation improves photosynthesis and growth in mustard through changes in proline accumulation and ethylene formation under drought stress. S Afr J Bot. 2015;98:84–94. 10.1016/j.sajb.2015.02.005.

[22] Ghassemi-Golezani K, Ghassemi S, Salmasi SZ. Changes in essential oil-content and composition of ajowan (*Carum copticum* L.) seeds in response to growth regulators under water stress. Sci Hortic. 2018;231:219–26. 10.1016/j.scienta.2017.12.011.

[23] El Tayeb MA, Ahmed NL. Response of Wheat Cultivars to Drought and Salicylic Acid. American-Eurasian Journal of Agronomy. 2010;3(1):01–7.

[24] Loutfy N, El-Tayeb MA, Hassanen AM, Moustafa MFM, Sakuma Y, Inouhe M. Changes in the water status and osmotic solute contents in response to drought and salicylic acid treatments in four different cultivars of wheat (*Triticum aestivum*). J Plant Res. 2012;125(1):173–84. 10.1007/s10265-011-0419-9. 21445718 10.1007/s10265-011-0419-9

[25] Chen YE, Cui JM, Li GX, Yuan M, Zhang ZW, Yuan S, Zhang HY. Effect of salicylic acid on the antioxidant system and photosystem II in wheat seedlings. Biol Plant. 2016;60(1):139–47. 10.1007/s10535-015-0564-4.

[26] Bandurska H, Stroi Ski A. The effect of salicylic acid on barley response to water deficit. Acta Physiol Plant. 2005;27(3):379–86. 10.1007/s11738-005-0015-5.

[27] Habibi G. Exogenous salicylic acid alleviates oxidative damage of barley plants under drought stress. Acta Biologica Szegediensis. 2012;56(1):57–63.

[28] Farooq M, Hussain M, Wahid A, Siddique KHM. Drought stress in plants: An overview. Plant Responses to Drought Stress: From Morphological to Molecular Features. 2012;9783642326530:1–33. 10.1007/978-3-642-32653-0_1.

[29] Saruhan N, Saglam A, Kadioglu A. Salicylic acid pretreatment induces drought tolerance and delays leaf rolling by inducing antioxidant systems in maize genotypes. Acta Physiol Plant. 2012;34(1):97–106. 10.1007/s11738-011-0808-7.

[30] Hayat S, Hasan SA, Fariduddin Q, Ahmad A. Growth of tomato (*Lycopersicon esculentum*) in response to salicylic acid under water stress. J Plant Interact. 2008;3(4):297–304. 10.1080/17429140802320797.

[31] Jumali SS, SIM, II, & ZZ. Genes Induced by High Concentration of Salicylic Acid in “*Mitragyna speciosa*.” Aust J Crop Sci. 2011: 5:296–303. https://search.informit.org/doi/10.3316/informit.27969482698072.

[32] Wani SH, Kumar V, Shriram V, Sah SK. Phytohormones and their metabolic engineering for abiotic stress tolerance in crop plants. Crop J. 2016;4(3):162–76. 10.1016/j.cj.2016.01.010.

[33] Munne-Bosch S, Penuelas J. Photo- and antioxidative protection, and a role for salicylic acid during drought and recovery in field-grown *Phillyrea angustifolia* plants. Planta. 2003;217(5):758–66. 10.1007/s00425-003-1037-0. 12698367 10.1007/s00425-003-1037-0

[34] Yalpani N, Enyedi AJ, León J, Raskin L. Ultraviolet light and ozone stimulate accumulation of salicylic acid, pathogenesis-related proteins and virus resistance in tobacco (Springer-Verlag). Planta. 1994;193(3):372–6.

[35] Sharma K, Fabre E, Tekotte H, Hurt EC, Tollervey D. Yeast Nucleoporin Mutants Are Defective in pre-tRNA Splicing. Mol Cell Biol. 1996;16(1):294–301. 10.1128/MCB.16.1.294. 8524308 10.1128/mcb.16.1.294 PMC231003

[36] Senaratna T, Touchell D, Bunn E, Dixon K. Acetyl salicylic acid (Aspirin) and salicylic acid induce multiple stress tolerance in bean and tomato plants. Plant Growth Regul. 2000;30(2):157–61. 10.1023/A:1006386800974.

[37] Larkindale J, Knight MR. Protection against Heat Stress-Induced Oxidative Damage in Arabidopsis Involves Calcium, Abscisic Acid, Ethylene, and Salicylic Acid. Plant Physiol. 2002;128(2):682–95. 10.1104/pp.010320. 11842171 10.1104/pp.010320 PMC148929

[38] Borsani O, Valpuesta V, Botella MA. Evidence for a Role of Salicylic Acid in the Oxidative Damage Generated by NaCl and Osmotic Stress in Arabidopsis Seedlings. Plant Physiol. 2001;126(3):1024–30. 10.1104/pp.126.3.1024. 11457953 10.1104/pp.126.3.1024 PMC116459

[39] Sallam A, Awadalla RA, Elshamy MM, Börner A, Heikal YM. Genome-wide analysis for root and leaf architecture traits associated with drought tolerance at the seedling stage in a highly ecologically diverse wheat population. Comput Struct Biotechnol J. 2024;23:870–82. 10.1016/j.csbj.2024.01.020. 38356657 10.1016/j.csbj.2024.01.020 PMC10864764

[40] Weatherley PE. Studies in the water relations of the cotton plant. New Phytol. 1950;49(1):81–97. 10.1111/j.1469-8137.1950.tb05146.x.

[41] El-Sharkawi HM, Salama FM. Effects of drought and salinity on some growth-contributing parameters in wheat and barley. Plant Soil. 1977;46(2):423–33. 10.1007/BF00010098.

[42] Bajji M, Kinet J-M, Lutts S. The use of the electrolyte leakage method for assessing cell membrane stability as a water stress tolerance test in durum wheat. Plant Growth Regul. 2002;36(1):61–70. 10.1023/A:1014732714549.

[43] Hedge, J., Hofreiter, B., Whistler, R. Carbohydrate Chemistry. Academic Press, New York, 1962. p. 17.

[44] Scarponi L, Perucci P. Effect of Some s-Triazine Herbicides on Phosphatases from Corn (*Zea mays*) Roots. Weed Sci. 1986;34(6):807–10. 10.1017/S0043174500067928.

[45] Bradford MM. A rapid and sensitive method for the quantitation of microgram quantities of protein utilizing the principle of protein-dye binding. Anal Biochem. 1976;72:248–54. 10.1006/abio.1976.9999. (PMID: 942051). 942051 10.1016/0003-2697(76)90527-3

[46] Sunkara SK, Khairy M, El-Toukhy T, Khalaf Y, Coomarasamy A. The effect of intramural fibroids without uterine cavity involvement on the outcome of IVF treatment: a systematic review and meta-analysis. Hum Reprod. 2010;25(2):418–29. 10.1093/humrep/dep396. 19910322 10.1093/humrep/dep396

[47] Baliyan S, Mukherjee R, Priyadarshini A, Vibhuti A, Gupta A, Pandey RP, Chang C-M. Determination of Antioxidants by DPPH Radical Scavenging Activity and Quantitative Phytochemical Analysis of *Ficus religiosa*. Molecules. 2022;27(4):1326. 10.3390/molecules27041326. 35209118 10.3390/molecules27041326 PMC8878429

[48] Aebi H. Catalase in vitro. Methods Enzymol. 1984;105:121–6. 10.1016/S0076-6879(84)05016-3. 6727660 10.1016/s0076-6879(84)05016-3

[49] Malik CP, Singh MB. Plant Enzymology and Histo Enzymology. New Delhi: Kalyani Publishers; 1980. p. 286.

[50] Cho YK, Ahn HK. Purification and characterization of polyphenol oxidase from potato: ii. inhibition and catalytic mechanism. J Food Biochem. 1999;23(6):593–605. 10.1111/j.1745-4514.1999.tb00588.x.

[51] Utz HF. PLABSTAT A Computer Program for Statistical Analysis of Plant Breeding Experiments Version 3A-Pre of 2005–08-16; Institute of Plant Breeding, Seed Science and Population Genetics. Stuttgart: University of Hohenheim; 1986.

[52] Sallam A, Alqudah AM, Dawood MFA, Baenziger PS, Börner A. Drought Stress Tolerance in Wheat and Barley: Advances in Physiology, Breeding and Genetics Research. Int J Mol Sci. 2019;20(13):3137. 10.3390/ijms20133137. 31252573 10.3390/ijms20133137 PMC6651786

[53] Mwadzingeni L, Shimelis H, Dube E, Laing MD, Tsilo TJ. Breeding wheat for drought tolerance: Progress and technologies. J Integr Agric. 2016;15(5):935–43. 10.1016/S2095-3119(15)61102-9.

[54] Takahashi F, Kuromori T, Urano K, Yamaguchi-Shinozaki K, and Shinozaki K. Drought Stress Responses and Resistance in Plants: From Cellular Responses to Long-Distance Intercellular Communication. Front Plant Sci. 2020:11. 10.3389/fpls.2020.556972. 10.3389/fpls.2020.556972 PMC7511591 33013974

[55] Takahashi F, Suzuki T, Osakabe Y, Betsuyaku S, Kondo Y, Dohmae N, Fukuda H, Yamaguchi-Shinozaki K, Shinozaki K. A small peptide modulates stomatal control via abscisic acid in long-distance signaling. Nature. 2018;556(7700):235–8. 10.1038/s41586-018-0009-2. 29618812 10.1038/s41586-018-0009-2

[56] Takahashi F, Hanada K, Kondo T, Shinozaki K. Hormone-like peptides and small coding genes in plant stress signaling and development. Curr Opin Plant Biol. 2019;51:88–95. 10.1016/j.pbi.2019.05.011. 31265991 10.1016/j.pbi.2019.05.011

[57] Liu J, Qiu G, Liu C, Li H, Chen X, Fu Q, Lin Y, Guo B. Salicylic Acid, a Multifaceted Hormone, Combats Abiotic Stresses in Plants. Life. 2022;12(6):886. 10.3390/life12060886. 35743917 10.3390/life12060886 PMC9225363

[58] Han Y, Chaouch S, Mhamdi A, Queval G, Zechmann B, Noctor G. Functional Analysis of Arabidopsis Mutants Points to Novel Roles for Glutathione in Coupling H_2_O_2_ to Activation of Salicylic Acid Accumulation and Signaling. Antioxid Redox Signal. 2013;18(16):2106–21. 10.1089/ars.2012.5052. 23148658 10.1089/ars.2012.5052 PMC3629853

[59] Kaya C, Ashraf M, Alyemeni MN, Corpas FJ, Ahmad P. Salicylic acid-induced nitric oxide enhances arsenic toxicity tolerance in maize plants by upregulating the ascorbate-glutathione cycle and glyoxalase system. J Hazard Mater. 2020;399: 123020. 10.1016/j.jhazmat.2020.123020. 32526442 10.1016/j.jhazmat.2020.123020

[60] Wang B, Song N, Zhang Q, Wang N, and Kang Z. TaMAPK4 Acts as a Positive Regulator in Defense of Wheat Stripe-Rust Infection. Front Plant Sci. 2018:9. 10.3389/fpls.2018.00152. 10.3389/fpls.2018.00152 PMC5829626 29527215

[61] Nyaupane S, Poudel MR, Panthi B, Dhakal A, Paudel H, and Bhandari R. Drought stress effect, tolerance, and management in wheat – a review. Cogent Food Agric. 2024:10(1). 10.1080/23311932.2023.2296094.

[62] Chaves MM, Flexas J, Pinheiro C. Photosynthesis under drought and salt stress: regulation mechanisms from whole plant to cell. Ann Bot. 2009;103(4):551–60. 10.1093/aob/mcn125. 18662937 10.1093/aob/mcn125 PMC2707345

[63] Kang G, Li G, Xu W, Peng X, Han Q, Zhu Y, Guo T. Proteomics Reveals the Effects of Salicylic Acid on Growth and Tolerance to Subsequent Drought Stress in Wheat. J Proteome Res. 2012;11(12):6066–79. 10.1021/pr300728y. 23101459 10.1021/pr300728y

[64] Sharma M, Gupta SK, Majumder B, Maurya VK, Deeba F, Alam A, Pandey V. Salicylic acid mediated growth, physiological and proteomic responses in two wheat varieties under drought stress. J Proteomics. 2017;163:28–51. 10.1016/j.jprot.2017.05.011. 28511789 10.1016/j.jprot.2017.05.011

[65] Manivannan PCA, Jaleel A, Kishorekumar B, Sankar R, Somasundaram R, Sridharan R. Drought stress induced changes in the biochemical parameters and photosynthetic pigments of cotton (*Gossypium hirsutum* L.). Indian J Appl Pure Biol. 2007;52:369–72.

[66] Liu F, Jensen CR, Shahanzari A, Andersen MN, Jacobsen S-E. ABA regulated stomatal control and photosynthetic water use efficiency of potato (*Solanum tuberosum* L.) during progressive soil drying. Plant Science. 2005;168(3):831–6. 10.1016/j.plantsci.2004.10.016.

[67] Akram S, Ghaffar M, Wadood A, Shokat S, Hameed A, Waheed MQ, and Arif MAR. A GBS-based genome-wide association study reveals the genetic basis of salinity tolerance at the seedling stage in bread wheat (*Triticum aestivum* L.). Front Genet. 2022:13. 10.3389/fgene.2022.997901. 10.3389/fgene.2022.997901 PMC9551609 36238161

[68] Kulkarni M, Soolanayakanahally R, Ogawa S, Uga Y, Selvaraj MG, Kagale S. Drought Response in Wheat: Key Genes and Regulatory Mechanisms Controlling Root System Architecture and Transpiration Efficiency. Front Chem. 2017;5:106. 10.3389/fchem.2017.00106. 29259968 10.3389/fchem.2017.00106 PMC5723305

[69] Kou X, Han W, and Kang J. Responses of root system architecture to water stress at multiple levels: A meta-analysis of trials under controlled conditions. Front Plant Sci. 2022:13. 10.3389/fpls.2022.1085409. 10.3389/fpls.2022.1085409 PMC9780461 36570905

[70] Reddy AR, Chaitanya KV, Vivekanandan M. Drought-induced responses of photosynthesis and antioxidant metabolism in higher plants. J Plant Physiol. 2004;161(11):1189–202. 10.1016/j.jplph.2004.01.013. 15602811 10.1016/j.jplph.2004.01.013

[71] Abdelaal KAA, Elafry M, Abdel-Latif I, Elshamy R, Hassan M, Hafez Y. Pivotal role of yeast and ascorbic acid in improvement the morpho-physiological characters of two wheat cultivars under water deficit stress in calcareous soil. Fresenius Environ Bull. 2021;30:2554–65.

[72] Allahverdiyev TI. Effect of drought stress on some physiological traits of durum (*Triticum durum* Desf.) and bread (*Triticum aestivum* L.) wheat genotypes. J Stress Physiol Biochem. 2015;11(1):29–38.

[73] Sharma S, Villamor JG, Verslues PE. Essential Role of Tissue-Specific Proline Synthesis and Catabolism in Growth and Redox Balance at Low Water Potential. Plant Physiol. 2011;157(1):292–304. 10.1104/pp.111.183210. 21791601 10.1104/pp.111.183210 PMC3165878

[74] Rashwan E, Alsohim AS, El-Gammaal A, Hafez Y, Abdelaal K. Foliar application of nano zinc-oxide can alleviate the harmful effects of water deficit on some flax cultivars under drought conditions. Fresenius Environ Bull. 2020;29:8889–904.

[75] Dawood MFA, Abeed AHA. Spermine-priming restrained water relations and biochemical deteriorations prompted by water deficit on two soybean cultivars. Heliyon. 2020;6(5): e04038. 10.1016/j.heliyon.2020.e04038. 32509989 10.1016/j.heliyon.2020.e04038 PMC7264753

[76] Shao RX, Xin LF, Guo JM, Zheng HF, Mao J, Han XP, Jia L, Jia SJ, Du CG, et al. Salicylic acid-induced photosynthetic adaptability of *Zea mays* L. to polyethylene glycol-simulated water deficit is associated with nitric oxide signaling. Photosynthetica. 2018;56(4):1370–137. 10.1007/s11099-018-0850-4.

[77] Khalvandi M, Siosemardeh A, Roohi E, Keramati S. Salicylic acid alleviated the effect of drought stress on photosynthetic characteristics and leaf protein pattern in winter wheat. Heliyon. 2021;7(1): e05908. 10.1016/j.heliyon.2021.e05908. 33490676 10.1016/j.heliyon.2021.e05908 PMC7809382

[78] Guo Z, Ou W, Lu S, Zhong Q. Differential responses of antioxidative system to chilling and drought in four rice cultivars differing in sensitivity. Plant Physiol Biochem. 2006;44(11–12):828–36. 10.1016/j.plaphy.2006.10.024. 17098438 10.1016/j.plaphy.2006.10.024

[79] Dudziak K, Zapalska M, Börner A, Szczerba H, Kowalczyk K, Nowak M. Analysis of wheat gene expression related to the oxidative stress response and signal transduction under short-term osmotic stress. Sci Rep. 2019;9(1):2743. 10.1038/s41598-019-39154-w. 30808876 10.1038/s41598-019-39154-w PMC6391441

[80] Espinoza A, San Martín A, López-Climent M, Ruiz-Lara S, Gómez-Cadenas A, Casaretto JA. Engineered drought-induced biosynthesis of α-tocopherol alleviates stress-induced leaf damage in tobacco. J Plant Physiol. 2013;170(14):1285–94. 10.1016/j.jplph.2013.04.004. 23651908 10.1016/j.jplph.2013.04.004

[81] Anwar T, Shehzadi A, Qureshi H, Shah MN, Danish S, Salmen SH, Ansari MJ. Alleviation of cadmium and drought stress in wheat by improving growth and chlorophyll contents amended with GA3 enriched deashed biochar. Sci Rep. 2023;13(1):18503. 10.1038/s41598-023-45670-7. 37898671 10.1038/s41598-023-45670-7 PMC10613229

[82] Ma J, Zhao D, Tang X, Yuan M, Zhang D, Xu M, Duan Y, Ren H, Zeng Q, Wu J, et al. Genome-Wide Association Study on Root System Architecture and Identification of Candidate Genes in Wheat (*Triticum aestivum* L.). Int J Mol Sci. 2022:23(3). 10.3390/ijms23031843. 10.3390/ijms23031843 PMC8836572 35163763

[83] Sankari M, Hridya H, Sneha P, Doss CGP, Christopher JG, Mathew J, Zayed H, Ramamoorthy S. Implication of salt stress induces changes in pigment production, antioxidant enzyme activity, and qRT-PCR expression of genes involved in the biosynthetic pathway of *Bixa orellana* L. Funct Integr Genomics. 2019;19(4):565–74. 10.1007/s10142-019-00654-7. 30694406 10.1007/s10142-019-00654-7

[84] Bowne JB, Erwin TA, Juttner J, Schnurbusch T, Langridge P, Bacic A, Roessner U. Drought Responses of Leaf Tissues from Wheat Cultivars of Differing Drought Tolerance at the Metabolite Level. Mol Plant. 2012;5(2):418–29. 10.1093/mp/ssr114. 22207720 10.1093/mp/ssr114

[85] Sara K, Abbaspour H, Sinaki JM, Makarian H. Effects of Water Deficit and Chitosan Spraying on Osmotic Adjustment and Soluble Protein of Cultivars Castor Bean. J Stress Physiol Biochem. 2012;8(3):160–9.

[86] Kabiri R, Nasibi F, Farahbakhsh H. Effect of Exogenous Salicylic Acid on Some Physiological Parameters and Alleviation of Drought Stress in *Nigella sativa* Plant under Hydroponic Culture. Plant Prot Sci. 2018;50:43–51.

[87] La VH, Lee B-R, Islam MdT, Park S-H, Jung H, Bae D-W, Kim T-H. Characterization of salicylic acid-mediated modulation of the drought stress responses: Reactive oxygen species, proline, and redox state in *Brassica napus*. Environ Exp Bot. 2019;157:1–10. 10.1016/j.envexpbot.2018.09.013.

[88] El-Esawi MA, Elashtokhy MMA, Shamseldin SAM, El-Ballat EM, Zayed EM, Heikal YM. Analysis of Genetic Diversity and Phylogenetic Relationships of Wheat (*Triticum aestivum* L.) Genotypes Using Phenological, Molecular and DNA Barcoding Markers. Genes (Basel). 2022;14(1):34. 10.3390/genes14010034. 36672774 10.3390/genes14010034 PMC9858705

[89] Ahmed K, Shabbir G, Ahmed M, Shah KN. Phenotyping for drought resistance in bread wheat using physiological and biochemical traits. Sci Total Environ. 2020;729: 139082. 10.1016/j.scitotenv.2020.139082. 32371202 10.1016/j.scitotenv.2020.139082 PMC7189857

[90] Hyles J, Bloomfield MT, Hunt JR, Trethowan RM, Trevaskis B. Phenology and related traits for wheat adaptation. Heredity (Edinb). 2020;125(6):417–30. 10.1038/s41437-020-0320-1. 32457509 10.1038/s41437-020-0320-1 PMC7784700

[91] Regmi S, Poudel B, Ojha BR, Kharel R, Joshi P, Khanal S, Kandel BP. Estimation of Genetic Parameters of Different Wheat Genotype Traits in Chitwan. Nepal International Journal of Agronomy. 2021;2021:1–10. 10.1155/2021/6651325.

[92] Narayanan S, Vara Prasad PV. Characterization of a Spring Wheat Association Mapping Panel for Root Traits. Agron J. 2014;106(5):1593–604. 10.2134/agronj14.0015.

[93] Lynch J. Root Architecture and Plant Productivity’. 1995;109:7. 10.1104/pp.109.1.7 PMC157559 12228579

[94] Waines JG, Ehdaie B. Domestication and Crop Physiology: Roots of Green-Revolution Wheat. Ann Bot. 2007;100(5):991–8. 10.1093/aob/mcm180. 17940075 10.1093/aob/mcm180 PMC2759207

[95] Sewore BM, Abe A, Nigussie M. Evaluation of bread wheat (*Triticum aestivum* L.) genotypes for drought tolerance using morpho-physiological traits under drought-stressed and well-watered conditions. PLoS One. 2023;18(5):e0283347. 10.1371/journal.pone.0283347. 37141261 10.1371/journal.pone.0283347 PMC10159169

