# Supplementary material for: The role of salicylic acid in modulating phenotyping in spring wheat varieties for mitigating drought stress
Source: BMC Plant Biol. 2024 Oct 11;24:948. doi: 10.1186/s12870-024-05620-5 (PMC11468136; doi:10.1186/s12870-024-05620-5)
Supplement: Supplementary file 1 — Supplementary Material 1. [file 12870_2024_5620_MOESM1_ESM.pptx]

## Slide 1
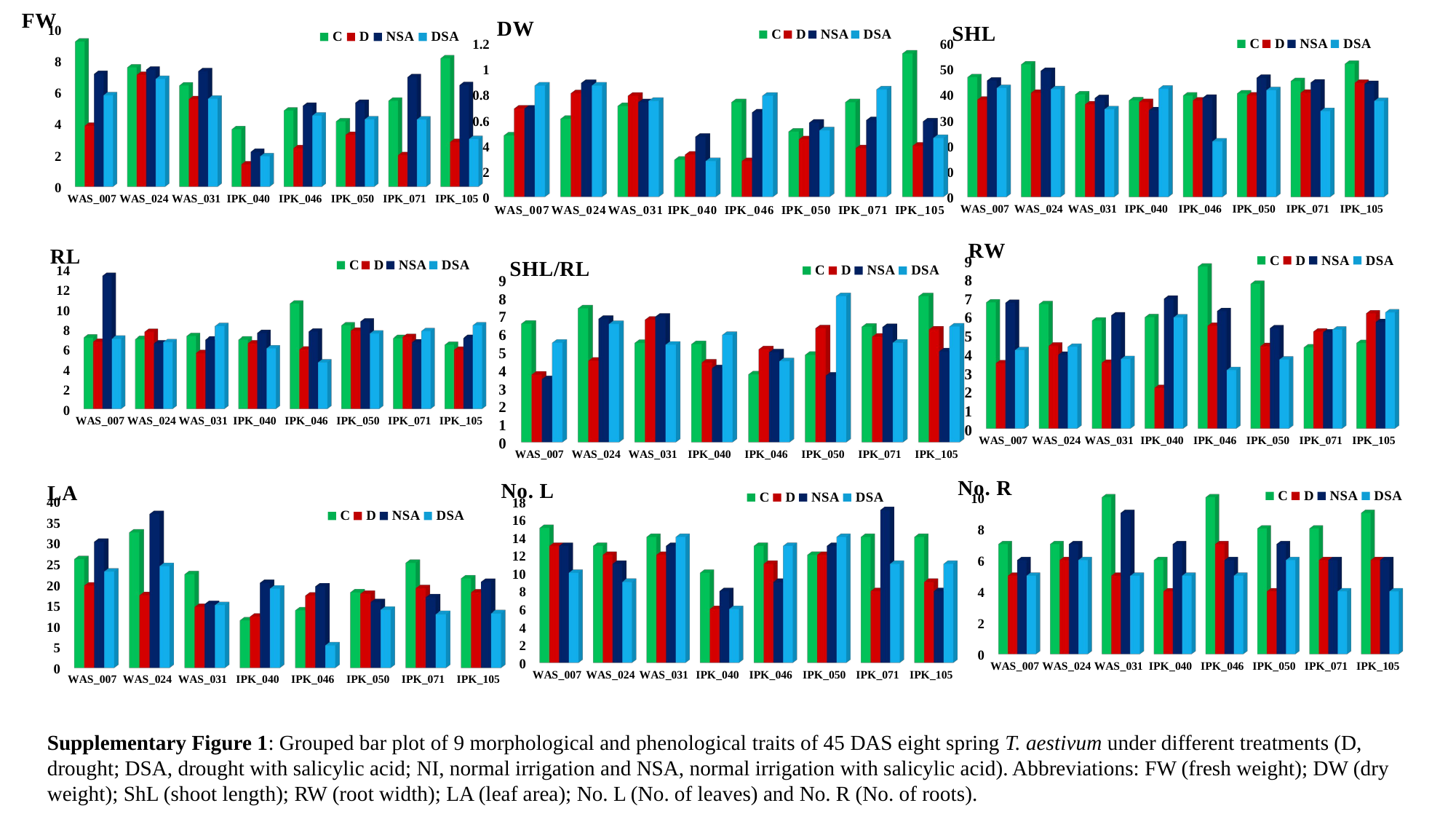

[unsupported chart]
[unsupported chart]
[unsupported chart]
[unsupported chart]
[unsupported chart]
[unsupported chart]
[unsupported chart]
[unsupported chart]
[unsupported chart]
Supplementary Figure 1: Grouped bar plot of 9 morphological and phenological traits of 45 DAS eight spring T. aestivum under different treatments (D, drought; DSA, drought with salicylic acid; NI, normal irrigation and NSA, normal irrigation with salicylic acid). Abbreviations: FW (fresh weight); DW (dry weight); ShL (shoot length); RW (root width); LA (leaf area); No. L (No. of leaves) and No. R (No. of roots).

## Slide 2
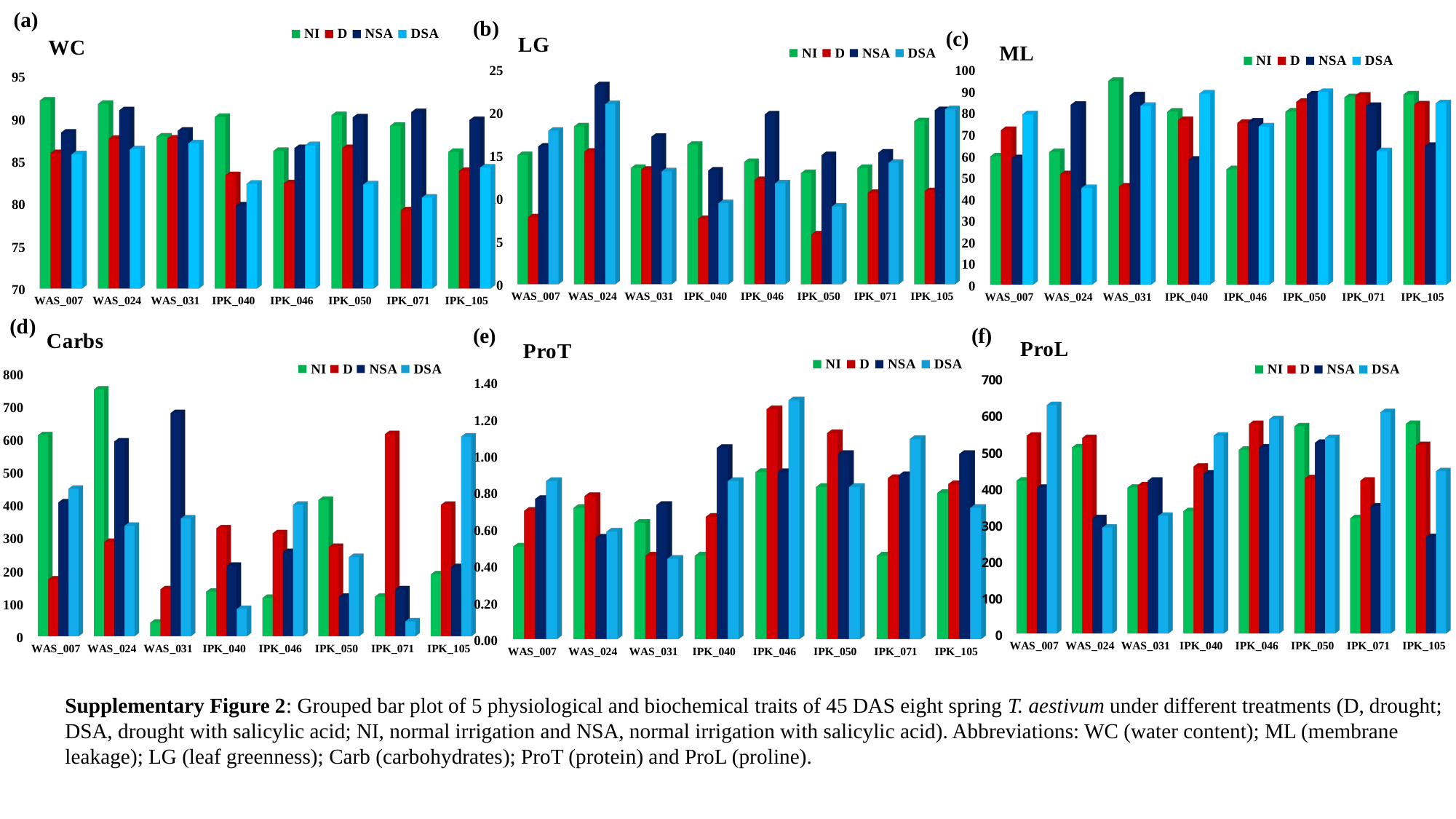

(a)
[unsupported chart]
[unsupported chart]
[unsupported chart]
[unsupported chart]
[unsupported chart]
[unsupported chart]
Supplementary Figure 2: Grouped bar plot of 5 physiological and biochemical traits of 45 DAS eight spring T. aestivum under different treatments (D, drought; DSA, drought with salicylic acid; NI, normal irrigation and NSA, normal irrigation with salicylic acid). Abbreviations: WC (water content); ML (membrane leakage); LG (leaf greenness); Carb (carbohydrates); ProT (protein) and ProL (proline).
